# The yeast stress inducible Ssa Hsp70 reduces α-synuclein toxicity by promoting its degradation through autophagy

**DOI:** 10.1371/journal.pgen.1007751

**Published:** 2018-10-30

**Authors:** Arpit Gupta, Anuradhika Puri, Prashant Singh, Surabhi Sonam, Richa Pandey, Deepak Sharma

**Affiliations:** G. N. Ramachandran Protein Centre, Council of Scientific and Industrial Research-Institute of Microbial Technology, Chandigarh, India; University of Massachusetts Amherst, UNITED STATES

## Abstract

The mechanism underlying the role of Hsp70s in toxicity associated with intracellular accumulation of toxic protein inclusions is under intense investigation. In current study, we examined the roles of all different isoforms of yeast cytosolic Ssa Hsp70 on α-synuclein mediated cellular toxicity. The study showed that yeast cells expressing stress-inducible Ssa3 or Ssa4 as sole Ssa Hsp70 isoforms, reduced α-synuclein toxicity better than those expressing a constitutive counterpart. The protective effect of stress-inducible Ssa Hsp70s was not α-syn specific, but more general to other inclusion forming proteins such as polyQ. We show that the protective effect is not by induction of a general stress response in Ssa3 cells rather by promoting α-synuclein degradation through autophagy. The present study revealed that effect of Hsp70s was isoform dependent, and that autophagy protects Ssa3 cells from the deleterious effects of toxic protein inclusions.

## Author summary

The accumulation of protein inclusions is a common feature in many neurodegenerative diseases. The Hsp70 chaperones has emerged as potent suppressor of toxicity caused by protein inclusions such as those formed of α-synuclein and polyQ. The underlying mechanism of Hsp70 mediated effect remains unclear, and it is believed that anti-aggregation activity of Hsp70 reduces protein inclusion mediated toxicity. In the present study, we examined the role of yeast cytosolic Ssa Hsp70 family of chaperones on α-synuclein mediated toxicity. We show that stress inducible Ssa Hsp70s are better than their constitutive counterparts to protect cells from both α-synuclein and polyQ toxicity. Our study reveals that the role of stress inducible Hsp70 in autophagy mediated degradation of α-synuclein protect cells from α-synuclein mediated toxicity.

## Introduction

Accumulation of protein inclusions is associated with various human diseases, such as neurodegenerative diseases and type 2 diabetes. The normally soluble proteins of unrelated sequences, such as Aβ in Alzheimer’s disease, α-synuclein (α-syn) in Parkinson’s disease (PD), and polyglutamine (polyQ) expanded huntingtin in Huntington’s disease (HD), form morphologically similar amyloids that possess a core structure of characteristic cross β-conformation [1, 2], [3], [4]. At the cellular level, these diseases share many key pathogenic features, such as induction of oxidative stress, mitochondrial damage, and proteasomal dysfunction, suggesting that the underlying mechanism of cellular toxicity may be similar in various amyloid-based disorders [5, 6]. As formation of protein inclusion involves conformational changes in proteins, various chaperones have been extensively studied for their role in the process. Among them, the Hsp70 family of proteins have emerged as promising candidates to protect cells from amyloid-associated toxicity.

The Hsp70 and Hsp90 family of proteins are important molecular chaperones required for the folding of most intracellular proteins. The chaperones promote protein folding; however, if the folding fails, substrates either become aggregated, or are degraded by the cellular degradation machinery. The ubiquitin-proteasome system (UPS) and autophagy are two major cellular degradation pathways, with autophagy being more specific for long-lived proteins or larger aggregates [7, 8]. In addition to their primary function of preventing protein aggregation, the role of chaperones in directing terminally misfolded proteins to either proteasome or autophagy is also well established [9], [10].

Chaperones of various families, such as small heat shock proteins, Hsp40, Hsp70, Hsp90, and Hsp100 have been shown to influence amyloid formation, and downstream cytotoxic effects [11]. Small heat shock proteins modulate amyloidosis, as seen by an increase in their expression level upon amyloid deposition, their association with amyloid deposits, and induction of cytoprotective effects such as defense against α-syn and polyQ associated toxicity [12, 13]. The overexpression of Hsp104 suppresses polyQ toxicity in both cellular and mouse models [14]. Hsp104, in coordination with Hsp70 chaperones, dissembles preamyloid α-syn oligomers, and shows a neuroprotective effect in rat PD models [15]. Hsp90 also interacts with α-syn, and modulates its amyloid-forming tendency [16]. Similarly, other chaperones, such as αB-crystalline, haptoglobin, α_2_-macroglobulin, and Hsp70, suppress oligomer toxicity by binding and converting the toxic oligomeric species into larger, benign aggregates [17, 18].

Hsp70 is among the most extensively studied chaperones for its role in amyloid related disorders. In *Drosophila*, mouse, and yeast models, overexpression of Hsp70 has cytoprotective effects against α-syn toxicity [19], [20], [21], [22]. Also, overexpression of heat shock transcription factor 1 (HSF1), a dominant transcription factor that increases the overall amount of Hsp70 protein, significantly reduces α-syn toxicity [23]. Consistent with its effects when overexpressed, partial inhibition of Hsp70 activity results in increased α-syn cytotoxicity [24]. The chaperoning action of Hsp70 on α-syn aggregation has been well studied and it has been shown that Hsp70 interacts with α-syn, and inhibits its ability to form amyloid fibrils [25]. It is believed that Hsp70 interacts with either the soluble or fibrillar forms of the amyloidogenic protein, and alters not only its assembly into fibrils, but also its associated cytotoxic properties [25], [26]. As for polyQ diseases, Hsp73 (Hsc70), in coordination with Hsp40, suppresses polyQ toxicity [27]. Although the protective effect of Hsp70s on amyloid toxicity has been extensively investigated, the underlying mechanism remains elusive.

The highly conserved Hsp70 is ubiquitously present in all organisms from bacteria to humans. Hsp70 contains of two domains; the nucleotide binding domain (NBD) and the substrate binding domain (SBD). The Hsp70 reaction cycle begins with ATP binding at the NBD, which facilitates substrate interaction with the SBD. The substrate binding, coupled with ATP hydrolysis, leads to further conformational changes in the Hsp70, which leads to the closing of the SBD in its C-terminal α-helical region known as lid. As intrinsic ATPase activity of Hsp70 is very low, other co-chaperones, such as the Hsp40 family of proteins, are required for ATPase activity. Nucleotide exchange factors assist in the exchange of ADP with ATP in order to begin another reaction cycle. The Hsp70 co-chaperones not only modulate Hsp70 activity, but also provide functional specificity to Hsp70 [28, 29].

All eukaryotic cells contain multiple members of cytosolic Hsp70s, which perform both overlapping and distinct functions. Among the six known human cytosolic Hsp70 chaperones, three are stress inducible, and all show tissue specific expression [30]. Understanding the functional relevance of different Hsp70 isoforms remains a challenge, and most studies have focused on Hsp70s from simpler eukaryotic systems like the yeast *Saccharomyces cerevisiae*. *S*. *cerevisiae* possesses members of the Ssa and Ssb subfamilies of cytosolic Hsp70. The Ssa subfamily consists of four homologous members, Ssa1-4, of which expression of at least one, regardless of the isoform, is crucial for growth. Ssa1 and Ssa2 are constitutively expressed, while Ssa3 and Ssa4 are stress inducible. Several lines of evidence suggest functional distinctions among these highly homologous Hsp70 members. For instance, the yeast prion URE3, which is formed from the native protein Ure2, is antagonized by Ssa1, but not Ssa2 [31]; however, Ssa2, and not Ssa1, weakens *PSI*^*+*^, another yeast prion [32]. Similarly, Ssa1 and Ssa2 differ in their action on clathrin uncoating, as well as on the degradation of the gluconeogenesis enzyme FBPase [33, 34]. These studies suggest that different Hsp70 isoforms may respond differently to the accumulation of protein inclusions. Indeed, it has been shown that upon heat shock, the stress-inducible Ssa members differ from the isoforms that are constitutively expressed when it comes to protecting cells against α-syn toxicity [22].

*S*. *cerevisiae* is well-established for modelling various amyloid-based neurodegenerative diseases such as PD and HD. Similar to observations made for neuronal cells, expression of α-syn or polyQ above a threshold level in *S*. *cerevisiae* leads to its aggregation into potentially toxic inclusion bodies [35]. In the present study, we examined the growth effects of α-syn and polyQ overexpression on yeast cells expressing individual Ssa Hsp70 isoforms. The strains expressing stress-inducible Ssa Hsp70s were less susceptible to the associated growth defect than their constitutively expressing counterparts. We further show that Ssa3 mediated cytoprotection was because of enhanced degradation of α-syn through autophagy.

## Results

### Ssa3 reduces α-syn toxicity

**Fig 1:**
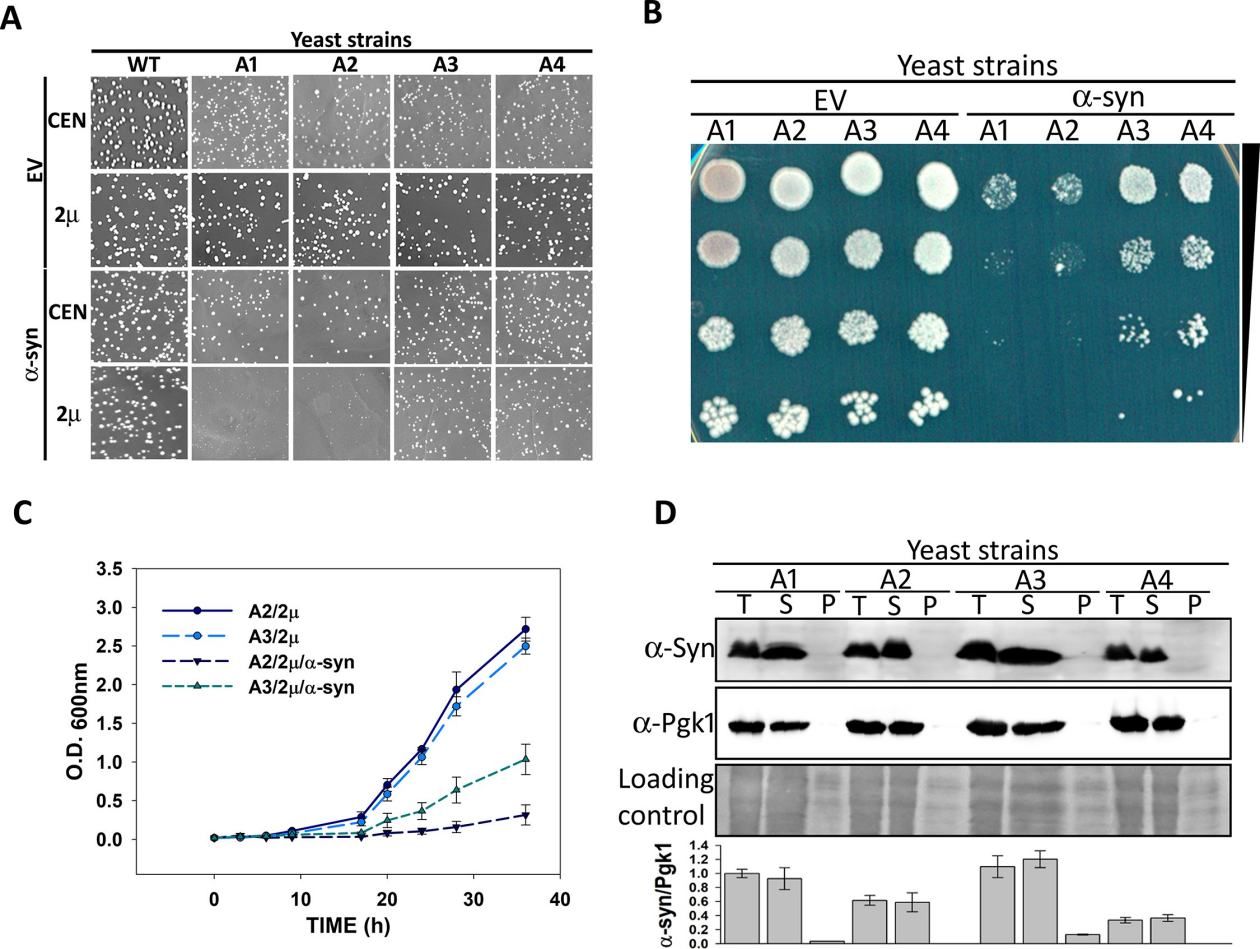
A3 strain reduced α-syn-mediated toxicity. The wt or strains A1-A4 were transformed with either CEN or 2μ plasmid, with or without the gene encoding α-syn, under the strong constitutive GPD promoter. **(A)** Growth phenotype of transformants after culturing 3 days at 30°C, and 2 days at 25°C. As seen, A1 and A2 grew slower than A3 and A4 upon α-syn overexpression from the 2μ plasmid. **(B)** Spot dilution assay to monitor the growth of strains transformed with 2μ plasmid, without (EV) or with the gene encoding α-syn. **(C)** Growth curve of indicated strains in liquid SD media lacking uracil. **(D)** Relative expression of α-syn from CEN plasmid in A1-A4 strains (top panel). The whole-cell lysate (T) was fractionated into soluble (S) and pellet (P) fractions. The proteins were separated on 12% SDS polyacrylamide gels and probed with anti α-syn antibodies. Middle and lower panels show loading controls. The bottom panel shows the relative quantification of α-syn levels, normalized to Pgk1 in strains A1-A4. Error bars represent standard error of replicates performed 3 times.

Hsp70 plays a key role in protecting cells from the toxic effects of protein inclusions; however, it is not clear how these aggregates are processed by the different Hsp70 isoforms. To discern the role of each Ssa Hsp70 isoform on α-syn mediated toxicity, we used yeast strains that expressed the desired isoform from a plasmid-borne gene transcribed from a constitutively expressing Ssa2 promoter, in the absence of all four chromosomally-encoded Ssa Hsp70s. For example, strains A1, A2, A3, and A4 expressed Ssa1, Ssa2, Ssa3, and Ssa4, respectively, in the absence of the remaining Ssa Hsp70 isoforms. The expression level of Ssa Hsp70 isoforms by the constructed strains was examined using immunoblotting and quantitative reverse transcriptase polymerase chain reaction (qRT-PCR) analyses, as described in the Materials and Methods. All Hsp70 isoforms were expressed at similar levels (S1 Fig). Strains A1-A4 were used to examine the effect of α-syn overexpression on cellular growth. The α-syn was expressed from either a centromeric (CEN) plasmid (pRS416P_GPD_-SYN) or 2μ plasmid (pRS426P_GPD_-SYN), using a strong constitutive glyceraldehyde-3-phosphate dehydrogenase (GPD) promoter. As seen in Fig 1A from similar colony size, when expressed from either CEN or 2μ plasmid, α-syn did not have a significant effect on the growth of wild-type (wt) cells encoding all four Ssa Hsp70 isoforms. However, overexpression of α-syn from the 2µ plasmid led to a severe growth defect for strains A1 and A2. Interestingly, the α-syn associated growth defect was much lower when Ssa3 or Ssa4 was used as the sole source of Ssa Hsp70, compared to Ssa1 or Ssa2. We further explored the effect of Ssa2 and Ssa3 as representative members of constitutive and stress-inducible Ssa Hsp70s, respectively, on α-syn toxicity. Fig 1B and 1C show the growth phenotypes of the strains overexpressing α-syn when cultured using solid and liquid media, respectively. As shown, the toxicity was significantly reduced in strains with the stress-inducible Ssa Hsp70 isoform. We also examined the effect of variation in Ssa Hsp70 expression on α-syn associated toxicity. The strains were constructed to express Ssa2 or Ssa3 from a CEN plasmid using Hsp82 promoter. In addition, expression level of Ssa Hsp70 was further increased by co-transforming two plasmids each encoding Ssa Hsp70 isoform under Ssa2 or Hsp82 promoter. As shown in S2 Fig, even at different expression levels, only cells expressing Ssa3 were able to reduce α-syn associated toxicity. Similar results were observed when α-syn or α-syn-GFP were induced using the galactose-inducible promoter in strains A2 and A3 (S3 Fig); however, the overall toxicity was less, compared to α-syn expressed from the GPD promoter in the 2μ plasmid. The wt strain harboring all Ssa Hsp70 isoforms show no significant difference upon α-syn overexpression (S4A Fig). Strains A3 and A4 are isogenic to strains A1 and A2 except for the presence of their respective stress-inducible Ssa Hsp70 isoforms. The above results reveal that the different Ssa Hsp70s, even if highly homologous, acted distinctly in response to the cytotoxic effects of α-syn. These findings are in agreement with previous reports suggesting that overexpression of Ssa3 reduces α-syn toxicity [22].

We next compared the relative expression of α-syn in wt as well as strains expressing one of the Ssa isoforms (A1-A4). The strains expressing α-syn from CEN-based plasmid with the GPD promoter were used to measure the protein levels. As shown in Fig 1D, the protein amounts were found to be similar from strains A1- A4, in both their whole-cell lysate, and their culture supernatants. Interestingly, the α-syn was found to be primarily in the soluble fraction of the whole-cell lysate, and not the pellet fraction. Similarly, the α-syn fractionated predominantly into the soluble fraction of the lysate from the wt strain (S4B and S4C Fig). As α-syn expressed at relatively lower level from CEN plasmid is non-toxic in A1-A4 strains, and primarily present in soluble fraction, the results suggests that at lower abundance the protein exists primarily in its non-toxic benign state. This is in agreement with a previous report that either a mutation of α-syn, or a two-fold overexpression caused by triplication of the α-syn encoding gene, is associated with cellular toxicity [36].

Because of enhanced toxicity in strains A1 and A2, we could not compare α-syn levels expressed from the 2µ plasmid with the GPD promoter. Therefore, using green fluorescent protein (GFP) as a reporter gene in place of α-syn, we measured the expression levels under similar conditions. As shown in S5 Fig, GFP was expressed similarly in strains A2 and A3. Overall, these results suggest that the reduced toxicity in strains A3 and A4 was not because of variations in the expression level of α-syn.

### Ssa3 reduces toxicity associated with α-syn mutants

**Fig 2:**
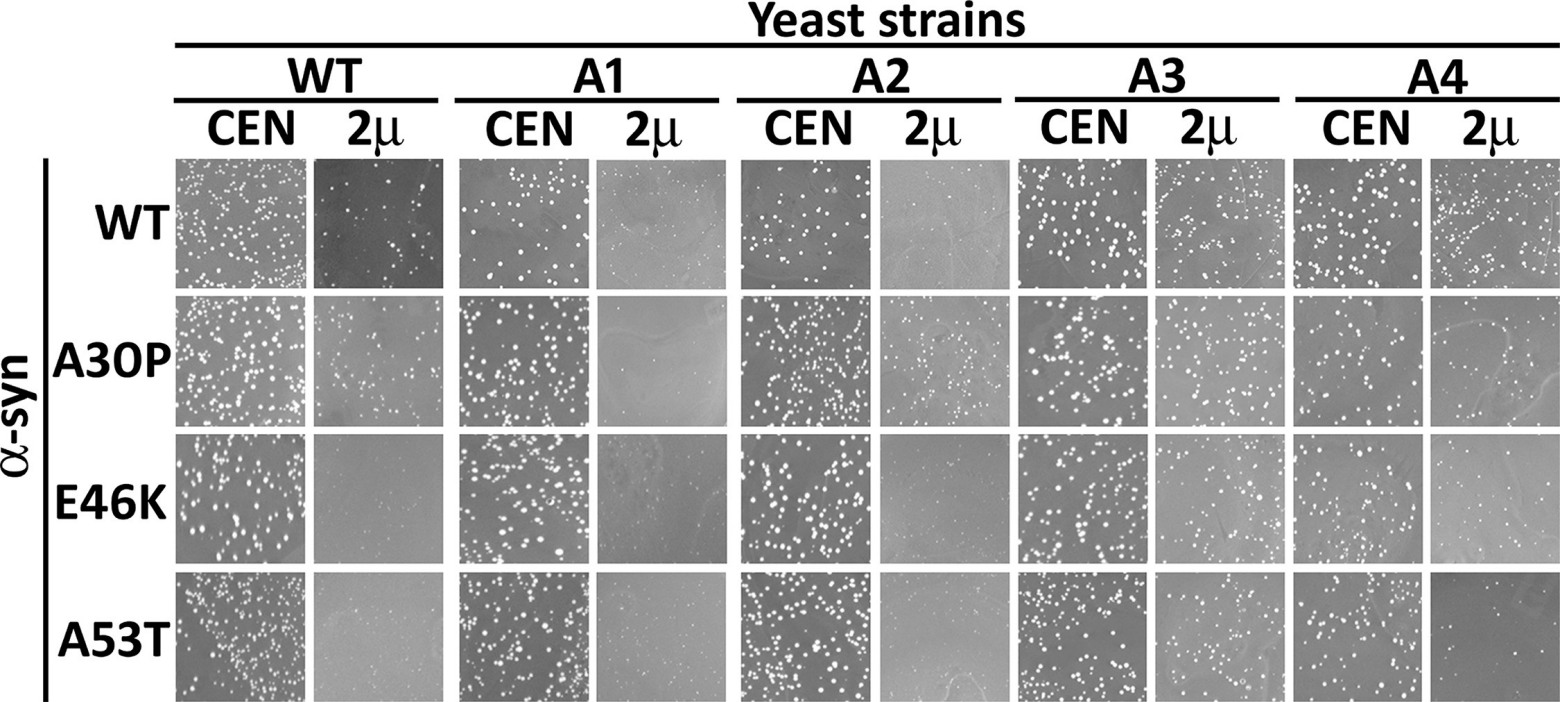
A3 and A4 strains reduced toxicity associated with α-syn mutants α-syn(A53T), α-syn(A30P), and α-syn(E46K). The indicated strains were transformed with centromeric (CEN) or 2µ plasmids encoding either wt, or one of the mutant α-syn genes under the strong GPD promoter. Shown is growth of transformants after incubation for 3 days at 30°C and 2 days at 25°C.

We next monitored the response of strains A3 and A4 against the toxicity associated with known disease-associated α-syn transition mutations, A30P, E46K and A53T. Among these three α-syn mutations, E46K and A53T are more fibrillogenic and toxic than A30P [37], [38],[39]. The growth phenotypes of these mutants were monitored as described above for the wt α-syn. As shown in Fig 2, the overexpression of each of the α-syn mutations led to growth defects in strain A1. For strain A2 the α-syn transition mutations E46K and A53T caused poor growth, while the α-syn A30P mutation was comparatively less toxic to cell growth. Similar to wt α-syn, the overexpression of these α-syn mutations had minimal effects in either A3 or A4 cell growth. Collectively, these results point towards a specific role for a stress-inducible cytosolic Hsp70 in counteracting α-syn associated toxicity.

### Ssa3 reduces toxicity associated with 72Q

It is known that α-syn toxicity is associated with its ability to form intracellular inclusions [40]. Similar intracellular cytotoxic inclusions are also formed by other intrinsically disordered proteins such as polyQ expanded huntingtin protein in HD [41, 42]. Therefore, we next examined whether the protective effects observed in strains A3 and A4 were specific against α-syn mediated toxicity, or are more general and also relevant to other, unrelated proteins that cause cellular toxicity through a similar mechanism. It is known that overexpression of a fragment of the huntingtin protein containing a polyQ stretch of approximately 72 (72Q) or more glutamines causes growth arrest in yeast [43]. We overexpressed FLAG tagged 72Q-CFP through a methionine regulatable promoter in wt strains or strains A1 through A4, and examined its effect on cell growth. No significant growth defect was observed upon expression of 72Q in wt cells (S6A Fig). Similar to as seen in case of α-syn, strains expressing stress-inducible Ssa3 or Ssa4, but not constitutive Ssa1 or Ssa2, reduced the level of 72Q-associated toxicity (S6B Fig). The expression level of 72Q was examined using dot blot analysis as previously described [44]. As seen in S6C Fig, 72Q was expressed at similar levels in all four strains (A1-A4). These dot blot results are in agreement with qRT-PCR findings that showed similar mRNA levels for 72Q-CFP in the different strains evaluated (S6D Fig). As strains A1 through A4 are isogenic except for the presence of the different Ssa isoforms, our studies using α-syn and 72Q suggest that stress-inducible Ssa Hsp70s may be more effective in protecting cells against toxicity associated with amyloidogenic protein-inclusions.

### Heat shock proteins Hsp104 and Hsp90 do not affect Ssa3-mediated reduction of α-syn toxicity

**Fig 3:**
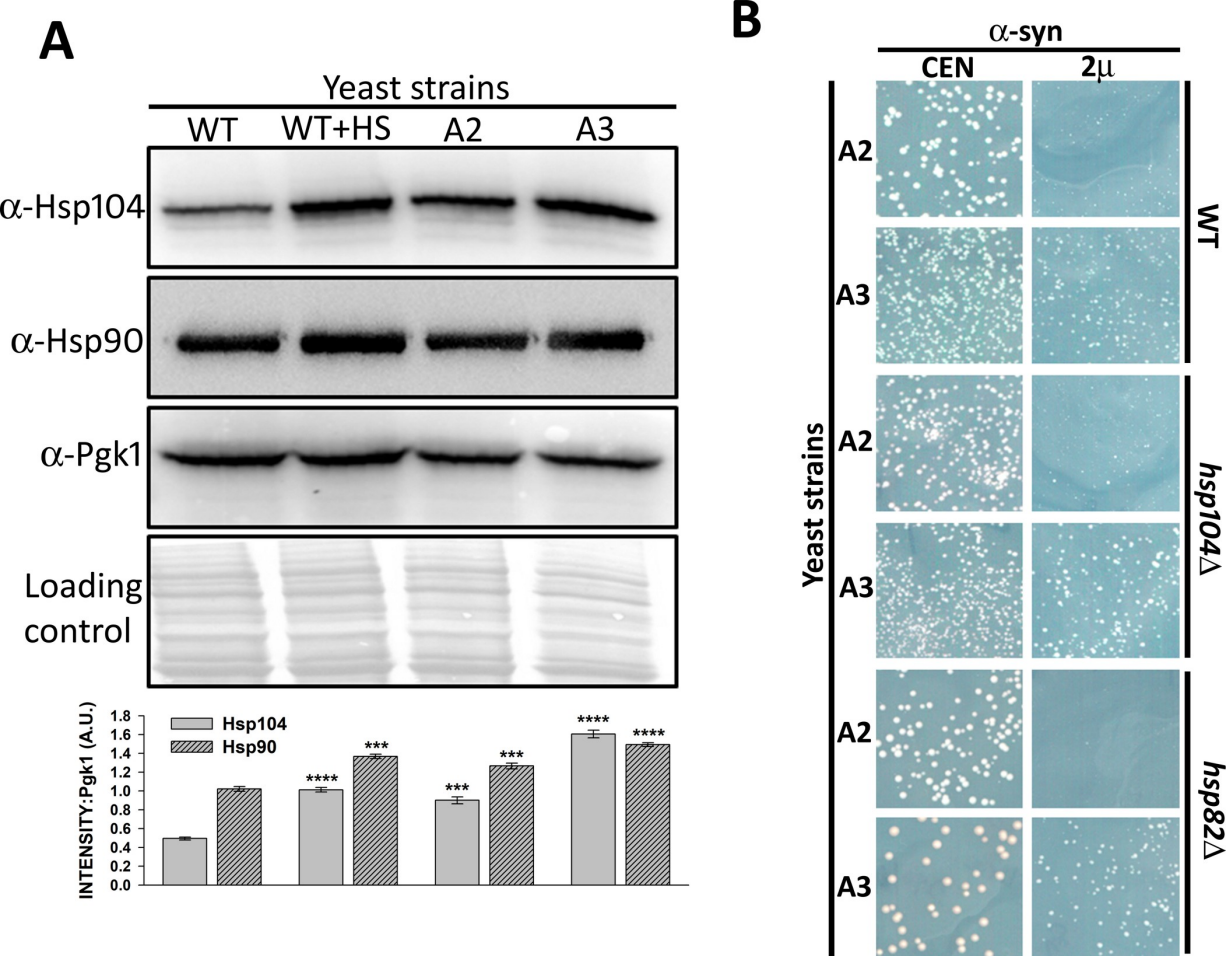
Reduction in α-syn toxicity in strain A3 was independent of elevated Hsp104 or Hsp90 levels. WT, A2, or A3 cells were grown in liquid media until mid-log phase. For heat shock, wt cells were incubated at 37°C for 30 minutes. **(A)** The whole-cell lysates were probed with anti-Hsp104, anti-Hsp90, or anti-Pgk1 (loading control). As seen, strain A3 showed elevated levels of Hsp90 and Hsp104, which was similar to wt cells following heat shock treatment. The bottom panel shows quantification of Hsp104 and Hsp90 levels, normalized to Pgk1. **(B)** The indicated strains were transformed with either CEN or 2µ plasmids encoding α-syn under the strong constitutive GPD promoter. Growth phenotypes were monitored after 3 days at 30°C and 2 days at 25°C. Averages from a minimum of three independent experiments are presented. Error bars represent the standard error. P-values were calculated using paired *t*-test using wt as a control.

The A3 strain lacks constitutive Ssa Hsp70 isoforms, which may lead to cellular stress. Therefore, we examined the levels of some of the chaperones that are known to be overexpressed under stress conditions. Indeed, the abundance of both Hsp104 and Hsp90 were found to be reproducibly higher in strain A3 than in strain A2 (Fig 3A). The increased amounts of Hsp104 relative to Hsp90 levels in strain A3 was much greater than what was observed in strain A2. This is in agreement with previous results showing a higher level of Hsp104 in the absence of Ssa1 and Ssa2 [45], [46]. Also, increase in both chaperones in strain A3 were similar to what was observed for wt strains under heat shock conditions. To explore whether the reduction in α-syn toxicity in strain A3 was due to increased levels of Hsp104 or Hsp90, we constructed A3 mutants that lacked either Hsp104, or inducible isoforms Hsp90, or Hsp82 [47]. As seen in Fig 3B, the overexpression of α-syn in strain A3 remained less toxic, even in the absence of Hsp104 or Hsp82, suggesting that increased levels of stress-inducible Hsp104 or Hsp90 were not the underlying mechanism of reduction in α-syn toxicity.

### The nucleotide binding domain governs functional distinctions between Ssa2 and Ssa3 with regard to α-syn toxicity

**Fig 4:**
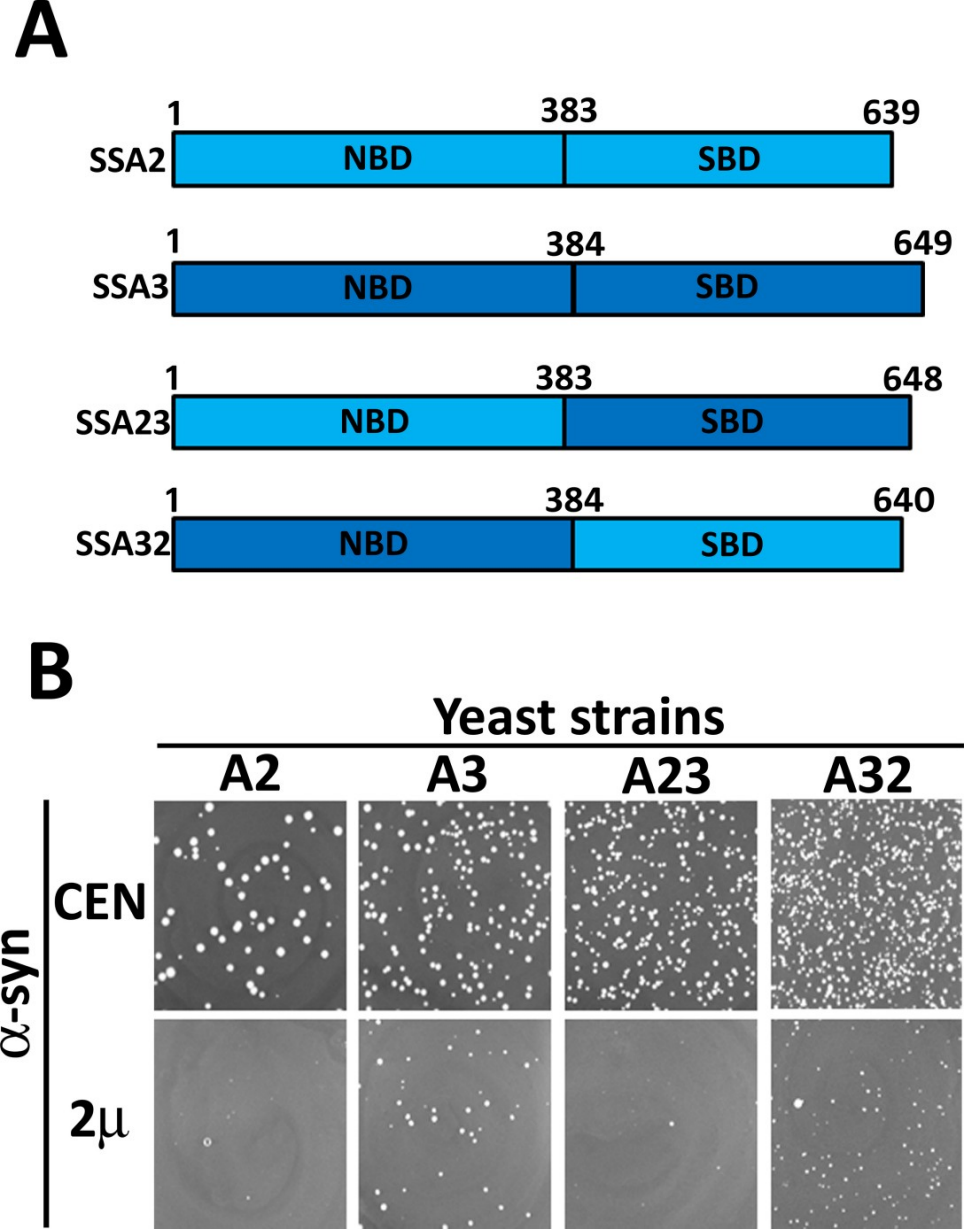
Nucleotide binding domain (NBD) regulated Hsp70 specificity against α-syn-mediated toxicity. **(A)** Schematic representation showing the domain architecture of Ssa2, Ssa3 and hybrid Ssa Hsp70s. **(B)** Growth phenotypes of indicated strains on solid selective SD media upon overexpression of α-syn. Growth was monitored as described in Fig 1A. Similar to strain A3, α-syn associated toxicity was reduced in strain A32.

The preceding findings suggest that stress-inducible Ssa Hsp70 chaperones functioned distinctly from their constitutive counterparts in reducing toxicity caused by the accumulation of α-syn or 72Q inclusions. To identify the Hsp70 structural domain that regulated this functional distinction, we further examined Ssa2 and Ssa3 as representative members of constitutive and stress-inducible Hsp70 isoforms, respectively. Based on Ssa2 and Ssa3 as parental proteins, hybrid proteins were constructed that possessed the nucleotide binding domain of one of the parental strains and the substrate binding domain from other (Fig 4A). For example, hybrid strain Ssa23 contained the nucleotide binding domain of Ssa2 and the substrate binding region of Ssa3. The strains expressing hybrid Ssa23 (A23) or Ssa32 (A32) as their only Ssa Hsp70 source were constructed and examined for their ability to counteract the α-syn mediated toxicity. In the absence of α-syn, strain A32 grew slower than those that expressed the parent protein, or the other hybrid (Ssa23), suggesting that while both designed hybrids could support yeast viability, Ssa32 was comparatively less capable of performing some of essential cellular function required for optimal cellular growth (S7 Fig). Fig 4B shows the effect of overexpression of α-syn from a CEN or 2μ-based plasmid with a GPD promoter on cell growth. Similar to what we observed for strain A2, overexpressed α-syn from the 2µ plasmid had a severe growth defect in A23. In the absence of α-syn, the strain expressing Ssa32 grew poorly compared to the Ssa23 expressing hybrid strain, but this growth phenotype was reversed upon overexpression of α-syn. This suggests that Ssa32, which contained the nucleotide binding domain of Ssa3, provided better cytoprotection against α-syn mediated toxicity. These results reveal an important role for the nucleotide binding domain in imparting the functional distinction between Ssa2 and Ssa3 in combating α-syn mediated toxicity.

### Autophagy impairment in A3 strain restores α-syn abundance and related toxicity

**Fig 5:**
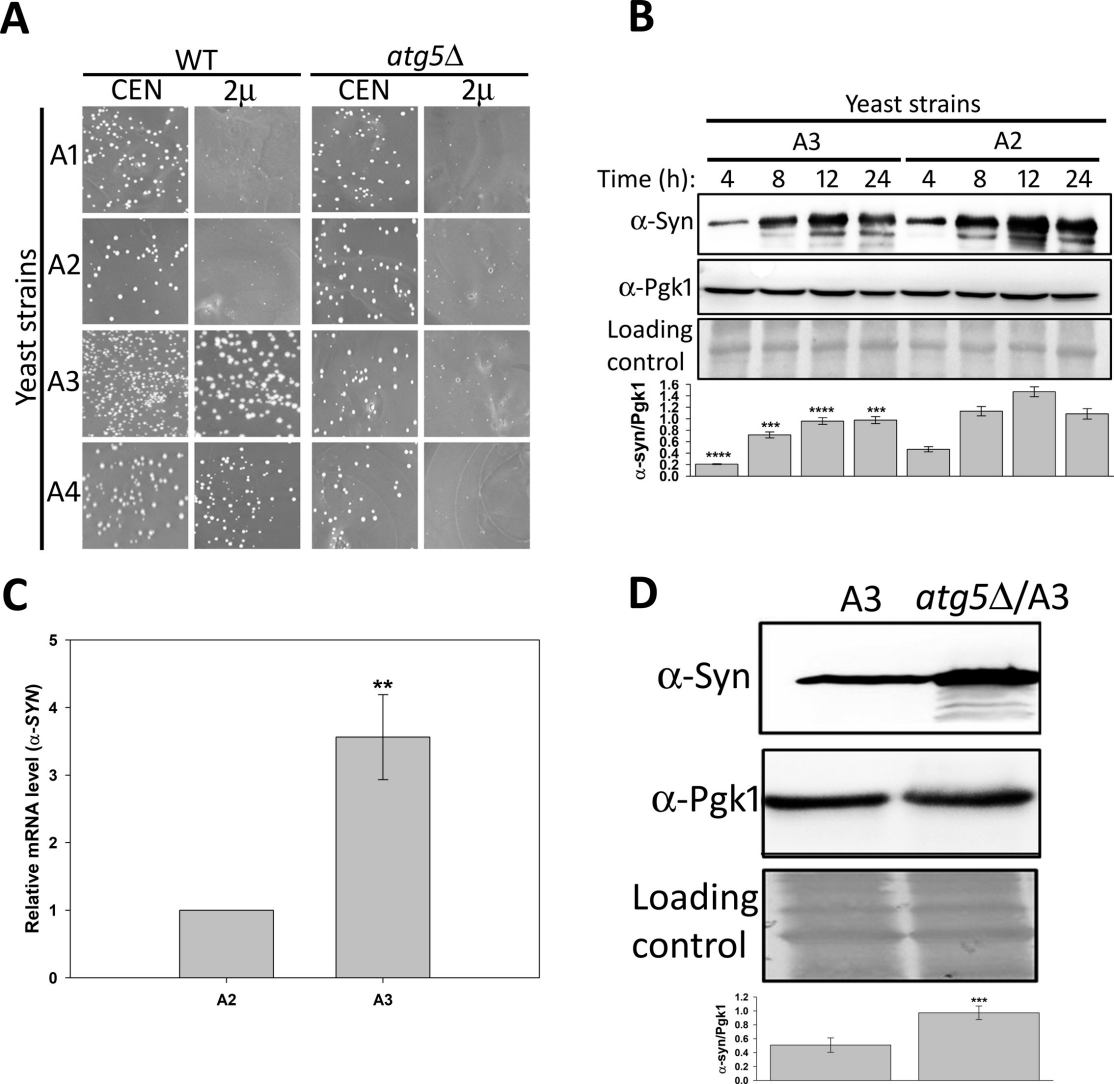
Autophagy inhibition enhanced α-syn-associated toxicity in strain A3. **(A)** The A1-A4 strains, which lack the gene encoding Atg5, were monitored for α-syn mediated toxicity as described above in Fig 1. In contrast to wt, the corresponding strains of A3 and A4 lacking Atg5 grew poorly upon α-syn overexpression. **(B)** The yeast strains transformed with plasmid encoding α-syn under a galactose inducible promoter were grown in inducing media. At indicated time, cells were collected, lysed, and the lysate probed with anti α-syn antibodies. The α-syn abundance was found to be lower in strain A3 than in strain A2 at all time points. **(C)** The indicated strains transformed with plasmid encoding α-syn under a galactose inducible promoter. Cells were grown under inducible conditions for 12 h. The qRT-PCR was carried out with primers specific for the genes encoding α-syn or Pgk1 (internal control). **(D)** The steady state level of α-syn was measured in whole-cell lysates obtained from the indicated strains. As seen, α-syn levels were up-regulated upon deletion of the Atg5 encoding gene (*atg5Δ*/A3). Error bars represent the standard error of replicates performed 3 times. P-values were calculated using paired *t*-test.

Hsp70s are also known to play a role in protein degradation. As α-syn is known to be an autophagic substrate, we explored whether autophagy modulated α-syn induced toxicity in strains A1 through A4. These strains were used as parental backbones to construct mutants lacking the gene that encodes Atg5. The *atg5Δ* mutants were examined for α-syn toxicity. A complex of Atg5 and Atg12 is required for the initiation of autophagosome formation, and thus the *atg5Δ* cells lacked normal autophagy activation. As seen in Fig 5A, α-syn overexpression in A1 or A2 strains lacking Atg5 resulted in growth defects that were similar to that observed for the corresponding wild-type strains. Interestingly, although cells that overexpressed α-syn demonstrated no significant growth defect in wild-type A3 or A4 strains, enhanced cellular toxicity was seen in the corresponding *atg5Δ* strains, indicating that Atg5 participates in a cellular process required for suppressing α-syn toxicity in strains that expressed inducible Ssa Hsp70s. We further examined the effect of *atg5Δ* on toxicity mediated by α-syn mutants in the A2 and A3 strains. Similar to results for wt α-syn, *atg5Δ* restored the growth defect phenotype of the α-syn mutants in strain A3 (S8 Fig).

As Atg5 is required for autophagy, and its deletion restored α-syn mediated toxicity, we explored whether the decreased toxicity in the A3 strain was because of increased degradation of α-syn through autophagy. We first compared the abundance of α-syn in strains A2 and A3 under conditions of mild cellular toxicity in the inducible yeast model. The α-syn expression was induced from a galactose inducible promoter, and culture aliquots were collected at regular time intervals. The α-syn level was determined in the cell lysates using immunoblotting with an anti-α-syn antibody. As seen in Fig 5B, α-syn was present at comparatively lower level in A3 than in A2 cells at all-time points examined. To determine whether lower steady-state levels of α-syn were because of reduced transcription, qRT-PCR from culture aliquots collected at 12 h was performed. The α-syn mRNA was found to be 2-3-fold higher in A3 compared to A2 cells (Fig 5C). Together, the immunoblotting and qRT-PCR data suggest that decreased levels of α-syn in strain A3 was not because of reduced transcription, and therefore could have been because of enhanced protein degradation. To examine whether autophagy was involved in degradation of α-syn in A3 strain, the protein’s steady-state level was monitored in the A3 strain lacking Atg5. As seen in Fig 5D, α-syn levels were increased by more than 2-fold upon deletion of the gene encoding Atg5, suggesting that inhibition of autophagy led to the impairment of α-syn degradation in strains expressing stress-inducible Ssa3.

### Autophagic induction and flux are higher in strain A3 than strain A2

**Fig 6:**
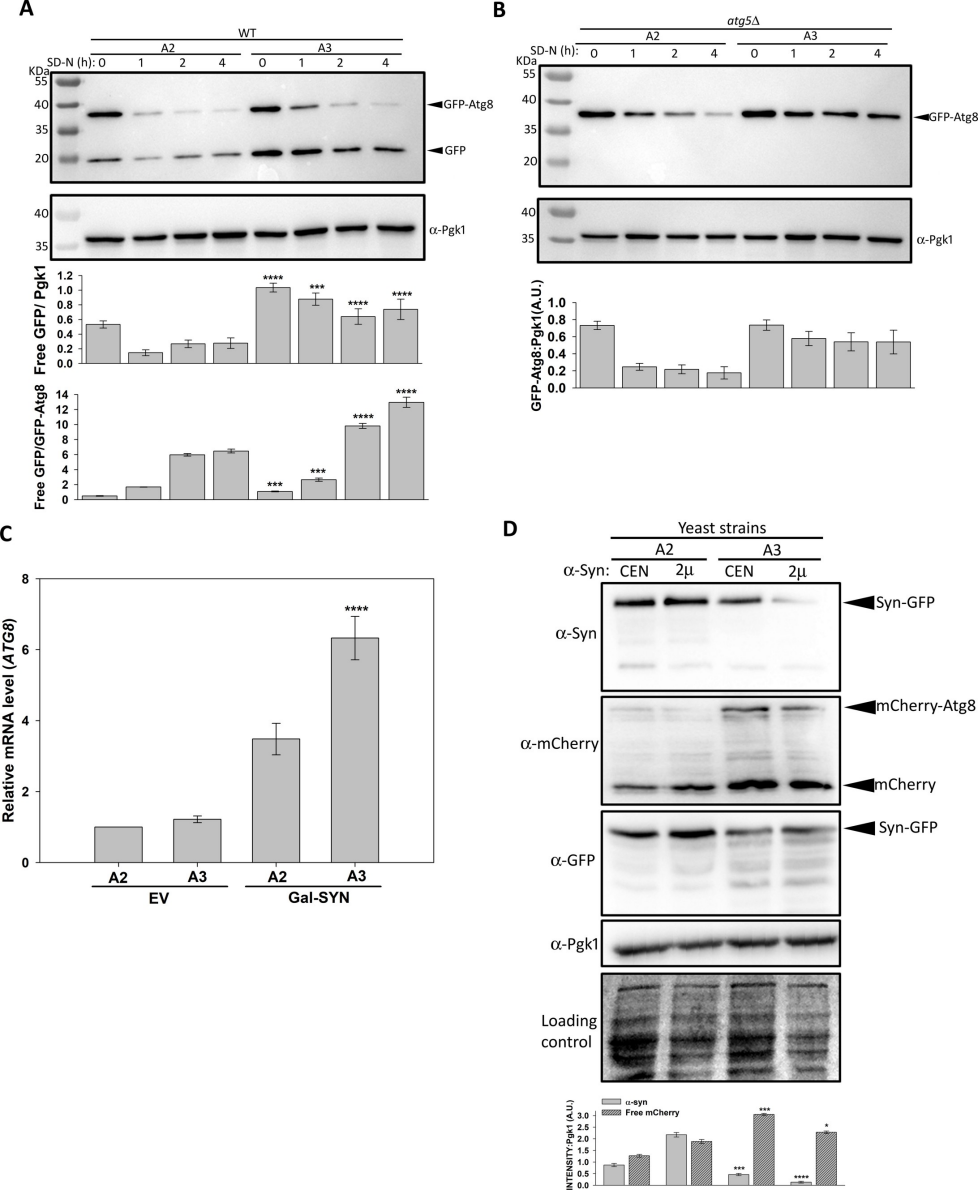
Strain A3 showed higher levels of autophagy upon α-syn expression. Strains A3, A4 **(A),** or corresponding strain *atg5Δ* **(B)** were grown in liquid SD media lacking a nitrogen source, and cellular lysates were probed with antibody against either GFP, or Pgk1 (loading control). The A3 strain showed increased processing of GFP-Atg8. Only unprocessed GFP-Atg8 was detected in the *atg5Δ* strain, as expected, because of the inhibition of autophagy. Immunoblotting using anti-Pgk1 antibody was used as a loading control. **(C)** Indicated strains transformed with empty plasmid, or plasmid encoding inducible α-syn, were grown in inducible media for 12 h. For quantitation, qRT-PCR was performed using primers specific for *ATG8*, or *PGK1* (internal control). As seen, α-syn mediated induction of Atg8 mRNA was higher in strain A3. **(D)** The plasmid encoding the mCherry-ATG8 fusion protein was co-transformed with p412P_GAL_-SYN-GFP (CEN) or p422P_GAL_-SYN-GFP (2μ) in strains A2 and A3. A pre-grown culture from SD media was diluted into SGal to a final concentration (O.D._600nm_) of 1.0, and further cultured for 12 h at 30°C. Cells were collected, and lysates probed with the indicated primary antibodies. Strain A3 showed reduced α-syn steady-state levels, and increased levels of free mCherry. The immunoblot with anti-GFP antibodies confirms relatively higher degradation of α-syn-GFP into various smaller fragments in A3 strain. Error bars represent the standard error of replicates performed 3 times. P-value were calculated using paired *t*-test.

We next examined how A2 and A3 strains responded to growth conditions that promoted autophagy induction. The fusion protein GFP-Atg8 is a widely used marker for autophagy [48]. The ubiquitin-like protein Atg8 localizes on the autophagosomal membrane through phospholipid phosphatidylethanolamine. Upon fusion of autophagosomes with vacuoles, Atg8 is proteolyzed by vacuolar proteases. However, GFP, which is relatively resistant, accumulates in the vacuoles [49]. Thus, the increase of GFP (from GFP-Atg8) accumulation in vacuoles corresponds to an increased autophagic flux. In our study, cells were grown in nitrogen deficient media to induce autophagy, and aliquots were collected at regular time intervals for immunoblot analysis using anti-GFP antibodies. S9 Fig shows increased amounts of free GFP when wt cells were used, as expected, suggesting increased autophagy flux under conditions of nitrogen starvation. Nitrogen starvation led to the release of free GFP in both A2 and A3 strains (Fig 6A), but the effect was more pronounced in strain A3. As expected, *atg5Δ* cells showed intact GFP-Atg8, with no free GFP; indicative of complete inhibition of autophagy (Fig 6B). The small decrease in GFP-Atg8 in *atg5Δ* cells could be due to its degradation through mechanism independent of autophagy. Also, A3 cells with the *atg5Δ* mutation showed higher steady-state levels of GFP-Atg8, which further indicated that autophagy activation by nitrogen starvation was stronger in the A3 cells.

To further explore the correlation between autophagy and α-syn degradation, we measured Atg8 transcript levels following the overexpression of α-syn. The mRNA levels of Atg8 were measured using qRT-PCR as described in Materials and Methods. We found that α-syn overexpression enhanced Atg8 transcription by approximately 3-fold in the wt strain (S10 Fig). In the absence of α-syn, Atg8 mRNA level was found to be similar in the strain A2 and A3 cells (Fig 6C). As shown, additional 3-fold and 6-fold increases in Atg8 transcription were observed upon α-syn induction in strains A2 and A3, respectively, suggesting that α-syn accumulation increased autophagy in both strains. This effect was even more pronounced in cells with Ssa3 as the sole Ssa Hsp70 source. Autophagy was further examined by monitoring free mCherry level in strains expressing mCherry-Atg8. Similar to findings from our nitrogen starvation experiments, compared to the corresponding A2 strain, A3 cells overexpressing α-syn-GFP showed higher free mCherry levels (Fig 6D). The absence of free mCherry from mCherry-Atg8 in *atg5Δ* strain confirms that the presence of free mCherry in otherwise wt strain is due to autophagy mediated degradation of Atg8 (S11 Fig). We also observed a corresponding decrease of α-syn levels with higher levels of free mCherry (Fig 6D, upper panel) in the A3 strain, suggesting a strong correlation of autophagy and α-syn clearance. Interestingly, instead of complete vacuolar mediated degradation of α-syn-GFP into free GFP, only partial degradation of the fusion protein was observed upon immunoblotting with anti-GFP antibody (Fig 6D, 3^rd^ panel). The incomplete processing of α-syn-GFP could be due to partial resistance of α-syn-GFP aggregates to degradation by vacuolar proteases.

**Fig 7:**
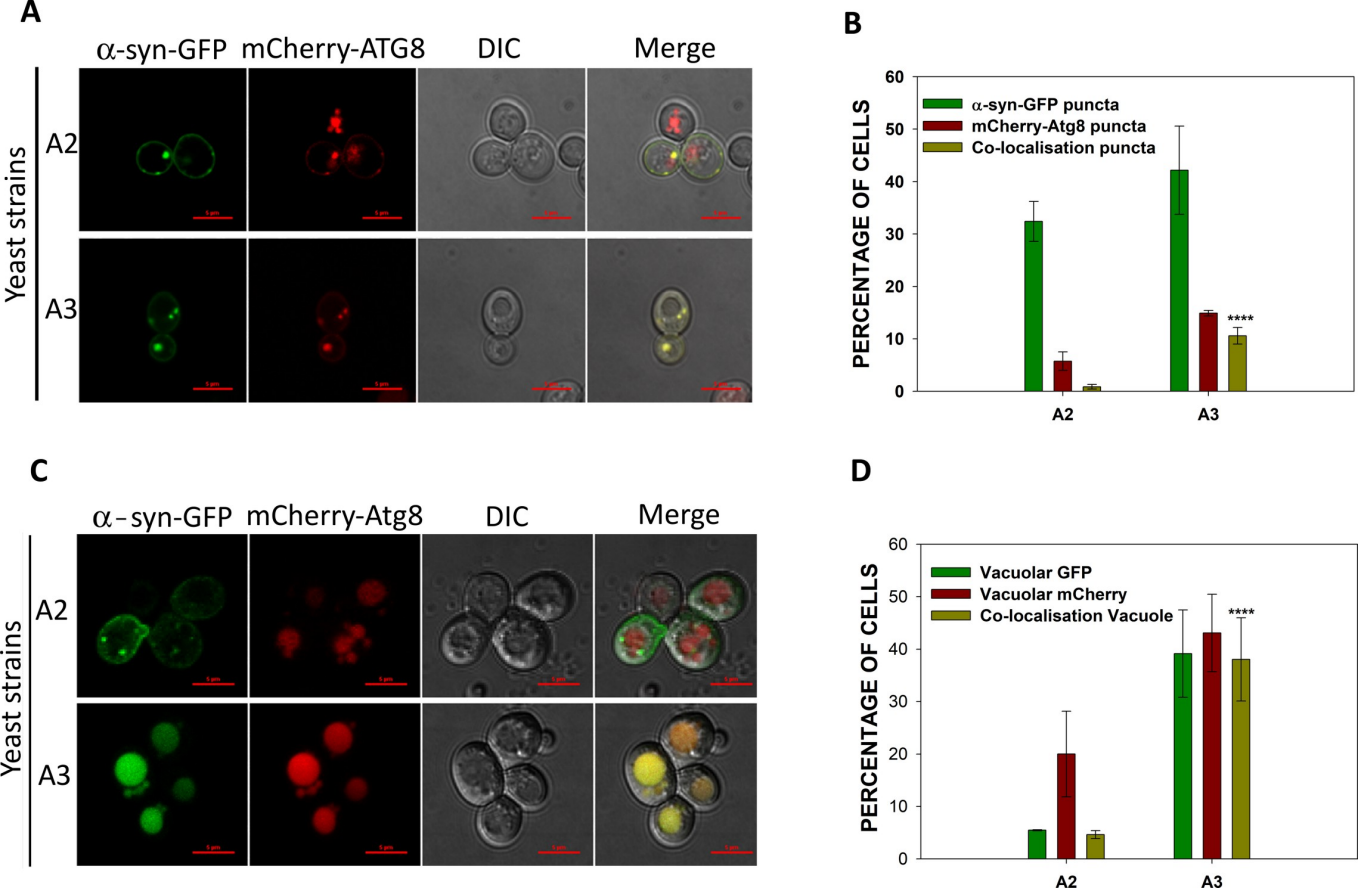
Co-localization of α-syn-GFP with autophagosomes, or vacuoles was higher in A3 than in A2 cells. Both strains A2 and A3 co-expressed α-syn-GFP and mCherry-Atg8. **(A)** Confocal microscopy images of representative cells. The α-syn-GFP expression was induced in galactose media for 12 h prior to collecting the cells for analysis by confocal microscopy. **(B)** The frequency of cells showing α-syn, mCherry, or co-localized puncta. **(C)** Representative confocal microscopy images of cells showing the presence of α-syn, or mCherry in vacuoles. **(D)** Frequency of cells showing vacuolar GFP or mCherry. Only those cells that showed GFP fluorescence were further examined for mCherry fluorescence. Error bars represent the standard error of more than 200 cells evaluated. P-value was calculated using paired *t*-test. Scale bar measures 5 μM.

Our studies were continued by measuring the accumulation of autophagosomes as a marker of autophagy. The autophagosomes were monitored by fluorescence-based localization of the mCherry-Atg8. Atg8 that is present on the surface of autophagosomal membranes appears as cytosolic puncta. Both A2 and A3 cells overexpressing α-syn-GFP were transformed with a plasmid encoding mCherry-Atg8. The cells were grown in inducible media for approximately 12 h, and visualized under fluorescence microscopy. Fig 7A shows the accumulation of α-syn inclusions (green puncta), autophagosomes (red puncta), and merged images of the two. Only cells that showed green fluorescence from α-syn-GFP were examined for the presence of autophagosomes. A slightly higher frequency of A3 cells containing green puncta were observed, suggesting more A3 cells contained α-syn aggregates (Fig 7B). Similarly, an increased number of A3 cells had autophagosomes compared to A2 cells (12% versus 5%, respectively), again in agreement with higher autophagy levels in A3 cells. Many of the α-syn inclusions co-localized with autophagosomes with the co-localization being significantly higher for A3 cells compared to A2 cells (9% versus 1%, respectively). Representative results are shown in Fig 7A and 7B. Cells with green fluorescence that also had mCherry in the vacuoles were used to estimate the autophagic flux (Fig 7C). Approximately 40% of the A3 cells showed this co-localization in vacuoles, compared to only 20% of A2 cells. Similarly, the frequency of A3 cells with vacuolar GFP fluorescence (40%) was significantly greater than that of A2 cells (7–8%), consistent with an increased autophagic flux of α-syn in the A3 cells (Fig 7D). We also compared α-syn-GFP localization in wt A3 and *atg5Δ*/A3 cells. As expected we observed increased number of α-syn-GFP puncta in A3 cells lacking Atg5 (S12 Fig). Overall, these results suggest that both increased autophagy induction and flux could have been responsible for the decreased α-syn level in A3 strain.

### Autophagy plays a dominant role over glutathione level in protecting A3 cells from α-syn mediated toxicity

It is known that accumulation of intracellular amyloids leads to increases in oxidative stress, which in turn contributes to cellular toxicity. To neutralize oxidative stress, cellular machinery has evolved a glutathione based system that maintains optimal redox potentials, and protects cells from deleterious consequences from increases in reactive oxygen species. We compared the reduced glutathione (GSH) levels in A2 and A3 cells, and the corresponding *atg5Δ* strains in efforts to determine the contribution of autophagy versus GSH. The cells were lysed, and equal amounts of lysate were measured for GSH levels as described in Materials and Methods. As shown in S13 Fig, A3 cells contained approximately 1.5-fold more GSH than A2 cells. The GSH levels increased significantly upon deletion of the Atg5 gene in both strains A2 and A3. An increased cellular toxicity in the A3 strain lacking Atg5, suggests that in the absence of autophagy, increased GSH level did not protect the cells from α-syn mediated toxicity. In contrast, GSH levels were lower in A3, yet they grew better than the A3/*atg5Δ* cells, again suggesting that autophagy played a dominant role over GSH in protecting cells from α-syn mediated toxicity and growth inhibition.

## Discussion

Amyloid deposition is a common feature in many neurodegenerative disorders such as PD, HD, and Alzheimer’s disease. Various cellular factors, primarily belonging to the family of chaperones, like Hsp70 proteins, are known to remodel amyloid fibrils [50, 51]. One aspect of Hsp70 function that has been extensively investigated is its ability to prevent protein aggregation, including those formed by amyloidogenic proteins such as α-syn. Studies show that Hsp70 is not only involved in the prevention of protein aggregation, but also in various other functions such as cell signaling, protein trafficking, and protein degradation, with many of these distinct processes being differentially regulated by closely related Hsp70 isoforms [52]. The present study shows that, although highly homologous, the constitutive and stress-inducible Ssa Hsp70s acted distinctly and differentially on protein inclusions, and that the protective effects of the stress-inducible Hsp70s against α-syn inclusions were by promoting α-syn degradation through autophagy.

We first examined how cells expressing individual Ssa Hsp70 isoforms responded to the overproduction of intrinsically disordered proteins such as wt α-syn, disease-associated α-syn mutants, and polyQ containing fragments of huntingtin. Interestingly, distinct cellular responses were observed in otherwise genotypically similar strains (A1-A4). Cells with stress-inducible Hsp70s showed reduced toxicity compared to those that were expressing constitutive isoforms. These results suggest that minor sequence variations imparted during evolution from constitutive counterparts to stress-inducible Ssa Hsp70s, were sufficient to discern distinct functional advantages in protecting cells from toxicity caused by protein inclusions. We found that when expressed at lower, non-toxic levels, the steady-state level of α-syn remained similar in strains A1 through A4, suggesting that the presence of different Hsp70 isoforms did not alter the stability of soluble α-syn. The reduced toxicity in A3 and A4 cells may have been because of their ability to cope with it through an appropriate cellular response. These findings are in agreement with those of a previous study that suggests the Ssa3 or Ssa4 is crucial for the protective effect of heat shock against α-syn toxicity [22]. Overall, these results suggest an important role for stress-inducible Hsp70s in protecting cellular viability against the toxicity associated with the accumulation of protein inclusions.

The increase in Hsp104 and Hsp90 levels in A3 cells may have been because of an induced heat shock response, resulting from the absence of constitutive Hsp70 isoforms Ssa1 and Ssa2. Lack of Hsp104 or inducible Hsp90 isoform had no effect on the reduction in toxicity in strain A3. Though the protective role of other stress inducible cellular factor could still not be ruled out these observations, along with previous findings that Ssa3 is essential for heat-stress mediated reduction in α-syn toxicity, suggests that elevated stress is unlikely to be sole basis of decreased toxicity.

Hybrid strains expressing Ssa Hsp70s revealed that the NBD, but not the SBD, imparted a functional advantage to Ssa3 against α-syn toxicity. It is interesting to note that hybrid strain A32 grew poorly than A23, but the overexpression of α-syn was more toxic to the A23 hybrid cells, suggesting that the rescue from protein-inclusion mediated toxicity required a highly specific action by Hsp70s, beyond its general role in maintenance of cellular homeostasis. It appears from our data that the regulation of Hsp70 activity is more important than the role of the substrate binding domain in interacting with α-syn. The significance of the NBD domain to govern functional distinction of Hsp70s has also been reported previously in various other studies [53], [54], [55], [56]. The activity of the NBD is regulated by interaction with various co-chaperones like Hsp40s and NEFs, many that are known to affect Hsp70s specificity [28]. Thus, interaction with a distinct co-chaperone, or differences in the binding affinity with a co-factor, may influence the ability of Ssa Hsp70 to respond to protein-inclusion mediated toxicity. Indeed, it has been shown that NEFs have specificity for Hsp70s, like Fes1 interacting specifically with Ssa, but not Ssb Hsp70s in vivo [57], [58]. Taken together, the NBD has a crucial role in regulating the functional specificity of Hsp70s.

The steady state level of inducible α-syn was found to be lower in strain A3 than strain A2. As the transcription of the gene encoding α-syn was not reduced in A3, the lower abundance may have been due to the enhanced degradation of the protein. The increase in the level of α-syn, and the restoration of toxicity in the mutant A3 strain that lacked Atg5 suggests that autophagy may be involved in α-syn degradation. These results are also in agreement with previous reports showing that α-syn is an autophagic substrate [7], [59], [60]. It is possible that Hsp70 chaperones are involved in the regulation of autophagy with different Hsp70 isoforms distinctly regulating the process. Alternatively, it is also possible that instead of a direct role for Hsp70 in autophagy regulation, the increased accumulation of α-syn inclusions in A3 strain upregulates autophagy as a protective cellular response. Furthermore, increased processing of GFP-Atg8 in A3 compared to A2 cells that lacked α-syn, suggests that A3 cells maintain a higher autophagic flux, even in the absence of α-syn inclusions. Ssa3 is a stress-inducible Hsp70 isoform, so the regulation of autophagy may be important in order to clear stress-mediated terminally damaged proteins that may not be refolded or degraded by other components of the cellular quality control system.

In the absence of α-syn, Atg8 transcript level was found to be similar in A2 and A3 cells. Interestingly, even under nitrogen containing growth medium, the overexpression of α-syn led to increased Atg8 transcription. The greater increase of *ATG8* in strain A3 than in strain A2 suggests that, while the response was higher in A3 cells, both A2 and A3 responded to α-syn toxicity by inducing autophagy. Atg8 was associated with autophagosomes, with the higher Atg8 levels correlating with a higher number of autophagosomes. The higher *ATG8* mRNA level in A3 strain correlated well with confocal microscopy results that the proportion of cells showing mCherry-Atg8 puncta was greater in strain A3 (16%) than in strain A2 (8%). The enhanced autophagy in A3 cells upon α-syn expression was further shown by increased processing of mCherry-Atg8. The increase of mCherry-Atg8 processing correlated with the decrease in α-syn levels, suggesting that enhanced autophagy was responsible for clearance of α-syn (Fig 6D). Overall, these results suggest that the enhanced autophagy in A3 cells following overexpression of α-syn may be involved in clearing α-syn aggregates, and thereby provide a protective response against toxicity mediated by α-syn inclusions. This is in agreement with previous findings that Ssa3 contributes to the removal of intracellular aggregates [61].

The confocal microscopy results showed that overexpression of α-syn led to its accumulation as cytosolic puncta; however, the number of cells with puncta was slightly higher in strain A3 (40%) than in strain A2 (32%). A smaller difference than expected in the frequency of cells with puncta may have been because of the enhanced autophagy in A3 cells that cleared the preformed puncta through vacuolar degradation. Indeed, the fraction of cells with green fluorescence (from GFP fused to α-syn) in the vacuoles was found to be significantly higher in strain A3 (40%) than in the isogenic strain A2 (5%). Since our immunoblot results (Fig 6D, 3^rd^ panel) with anti-GFP antibody shows only partial degradation of the fusion protein α-syn-GFP, the presence of green fluorescence in vacuoles is primarily through these partially degraded α-syn fragments fused to intact GFP. Similarly, cells containing vacuolar mCherry which is indicative of autophagic flux, were more prevalent in strain A3 than in strain A2. The increase in the number of A3 cells with mCherry co-localized with vacuolar GFP signal supports the conclusion that the increased autophagy contributed to the enhanced degradation of α-syn.

The glutathione assay showed that free GSH levels were relatively higher in cells positive for Ssa3 than in cells positive for Ssa2. The reduced form of glutathione (GSH) is known to scavenge free oxygen radicals, and thereby help protect cells from increased oxidative stress. It is possible that decreased toxicity in strain A3 was because of an increase in free GSH. However, increased free glutathione was not the sole underlying mechanism of reduced toxicity because the isogenic *atg5Δ* mutant had an even higher free glutathione level, but grow poorly. As there was a restoration of toxicity in the A3 strain lacking Atg5, despite a relatively higher glutathione level, autophagy may be the primary basis for reduction of α-syn mediated toxicity in strain A3.

The underlying mechanism of Ssa3 mediated activation of autophagy remains to be determined, but may be because of its direct or indirect role in autophagy. The results from hybrid Ssa Hsp70s point towards the role of interacting co-chaperones of the NBD of Ssa Hsp70s in reduction of α-syn associated toxicity. Autophagy is an evolutionarily conserved process that is upregulated under conditions of starvation or stress. As the stress-inducible Hsp70s participates in various cellular processes, including those required for the stress response, it is possible that Ssa3, in coordination with its co-chaperones, assisted in the maturation or activation of some of the key proteins involved in autophagy pathway.

All cells continually face the challenge to keep proteins in their functionally active, competent states. The challenges become even bigger under conditions of stress. To cope with stress, the cellular machinery upregulates various Hsp70 chaperones that function to maintain the integrity of the entire proteome. While the effects of constitutive members of chaperones are well studied, the stress-inducible counterpart is largely a mystery and thus, the reason cells require additional members in order to cope with stress remains unknown. Here, we have shown that stress-inducible Ssa3, which is upregulated under conditions of stress, have evolved mechanisms to remove toxic protein-inclusions through autophagy. Whether the results obtained using Ssa3 are extendable to Ssa4 or other stress-inducible remains to be determined. However, it is clear that the action of inducible Ssa Hsp70 chaperones is essential for cells to purge terminally misfolded proteins that upon accumulation become deleterious to cellular health. Though the current study reveals functional distinctions among yeast Ssa Hsp70s, there is little reported evidence that suggests that these findings might be extendable to mammalian Hsp70s. The human cytosolic Hsp70 is composed of six different isoforms, among which one is constitutive and the others inducible, or tissue specific. It has been shown that Hsp70 is involved in α-syn mediated degradation though autophagy [62]. Previous reports suggest a role for the heat shock response, as well as inducible Hsp70s, in regulating autophagy; however, whether a specific Hsp70 isoform is more effective than the other in autophagic process remains to be determined [63, 64]. The findings obtained in the present study warrant a comparative examination of the role of constitutive versus stress-inducible human Hsp70 isoforms in protein-misfolding disorders.

## Materials and methods

### Strains

The strains used in this study are listed in Table 1. The wild-type strain SY187 (WT) encodes all four Ssa Hsp70 isoforms. Strains SY135 (A1), SY136 (A2), SY143 (A3), and SY211 (A4) harbors plasmid pRS315PSSA2-SSA1/SSA2/SSA3/SSA4 that expresses Ssa1, Ssa2, Ssa3, or Ssa4 respectively as a sole source of Ssa Hsp70 isoform. These strains lack chromosomally encoded Ssa1-Ssa4; instead the desired Ssa Hsp70 isoform is expressed from a centromeric plasmid (CEN) under the constitutive Ssa2 promoter for their similar levels of expression. For construction of Atg5 knock-out strains, the gene encoding KanMX4 in SY135 was replaced with NAT to confer resistance against Nourseothricin. The resultant Nourseothricin resistant strain was further used to replace the Atg5 encoding gene with the KanMX4 by homologous recombination. Similarly, Hsp82 and Hsp104 knock-out strains were also constructed. Strains H32, H23, Asc200, ASc300, AY13, and AY14 were constructed by plasmid shuffling using strain SY135.

**Table 1:** List of strains.

Strains	Genotype	Reference
SY187	*MATa*, *kar 1–1*, *SUQ5*, *P*_*DAL5*_::*ADE2*, *his3Δ202*, *leu2Δ1*, *trp1Δ63*, *ura3-52*, [URE3]	Sharma *et al*, 2011
SY135	*MATa*, *P*_*DAL5*_::*ADE2*, *ssa1*::*Kan*, *ssa2*::*HIS3*, *ssa3*::*TRP1*, *ssa4*:: *ura3-2f/pRS315P*_*SSA2*_*-SSA1*	Sharma *et al*, 2008
SY136	*MATa*, *P*_*DAL5*_::*ADE2*, *ssa1*::*Kan*, *ssa2*::*HIS3*, *ssa3*::*TRP1*, *ssa4*:: *ura3-2f/pRS315P*_*SSA2*_*-SSA2*	Sharma *et al*, 2008
SY143	*MATa*, *P*_*DAL5*_::*ADE2*, *ssa1*::*Kan*, *ssa2*::*HIS3*, *ssa3*::*TRP1*, *ssa4*:: *ura3-2f/pRS315P*_*SSA2*_*-SSA3*	Sharma *et al*, 2008
SY211	*MATa*, *P*_*DAL5*_::*ADE2*, *ssa1*::*Kan*, *ssa2*::*HIS3*, *ssa3*::*TRP1*, *ssa4*:: *ura3-2f/pRS315P*_*SSA2*_*-SSA4*	Sharma *et al*, 2008
PPY222	*MATa*, *P*_*DAL5*_::*ADE2*, *hsp104*::*Kan*, *ssa1*::*Nat*, *ssa2*::*HIS3*, *ssa3*::*TRP1*, *ssa4*:: *ura3-2f/pRS315P*_*SSA2*_*-SSA2*	This study
PPY223	*MATa*, *P*_*DAL5*_::*ADE2*, *hsp104*::*Kan*, *ssa1*::*Nat*, *ssa2*::*HIS3*, *ssa3*::*TRP1*, *ssa4*:: *ura3-2f/pRS315P*_*SSA2*_*-SSA3*	This study
DD109	*MATa*, *P*_*DAL5*_::*ADE2*, *hsp82*::*Kan*, *ssa1*::*Nat*, *ssa2*::*HIS3*, *ssa3*::*TRP1*, *ssa4*:: *ura3-2f/pRS315P*_*SSA2*_*-SSA2*	This study
DD110	*MATa*, *P*_*DAL5*_::*ADE2*, *hsp82*::*Kan*, *ssa1*::*Nat*, *ssa2*::*HIS3*, *ssa3*::*TRP1*, *ssa4*:: *ura3-2f/pRS315P*_*SSA2*_*-SSA3*	This study
SY321	*MATa*, *P*_*DAL5*_::*ADE2*, *atg5*::*Kan*, *ssa1*::*Nat*, *ssa2*::*HIS3*, *ssa3*::*TRP1*, *ssa4*:: *ura3-2f/pRS315P*_*SSA2*_*-SSA1*	This study
SY322	*MATa*, *P*_*DAL5*_::*ADE2*, *atg5*::*Kan*, *ssa1*::*Nat*, *ssa2*::*HIS3*, *ssa3*::*TRP1*, *ssa4*:: *ura3-2f/pRS315P*_*SSA2*_*-SSA2*	This study
SY323	*MATa*, *P*_*DAL5*_::*ADE2*, *atg5*::*Kan*, *ssa1*::*Nat*, *ssa2*::*HIS3*, *ssa3*::*TRP1*, *ssa4*:: *ura3-2f/pRS315P*_*SSA2*_*-SSA3*	This study
SY324	*MATa*, *P*_*DAL5*_::*ADE2*, *atg5*::*Kan*, *ssa1*::*Nat*, *ssa2*::*HIS3*, *ssa3*::*TRP1*, *ssa4*:: *ura3-2f/pRS315P*_*SSA2*_*-SSA4*	This study
A32	*MATa*, *P*_*DAL5*_::*ADE2*, *ssa1*::*Kan*, *ssa2*::*HIS3*, *ssa3*::*TRP1*, *ssa4*:: *ura3-2f/pRS315P*_*SSA2*_*-SSA32*	This study
A23	*MATa*, *P*_*DAL5*_::*ADE2*, *ssa1*::*Kan*, *ssa2*::*HIS3*, *ssa3*::*TRP1*, *ssa4*:: *ura3-2f/pRS315P*_*SSA2*_*-SSA23*	This study
ASc100	*MATa*, *P*_*DAL5*_::*ADE2*, *ssa1*::*Kan*, *ssa2*::*HIS3*, *ssa3*::*TRP1*, *ssa4*:: *ura3-2f/pRS416P*_*GPD*_*-His*_*6*_*-SSA1*	This study
ASc200	*MATa*, *P*_*DAL5*_::*ADE2*, *ssa1*::*Kan*, *ssa2*::*HIS3*, *ssa3*::*TRP1*, *ssa4*:: *ura3-2f/pRS416P*_*GPD*_*-His*_*6*_*-SSA2*	This study
ASc300	*MATa*, *P*_*DAL5*_::*ADE2*, *ssa1*::*Kan*, *ssa2*::*HIS3*, *ssa3*::*TRP1*, *ssa4*:: *ura3-2f/pRS416P*_*GPD*_*-His*_*6*_*-SSA3*	This study
ASc400	*MATa*, *P*_*DAL5*_::*ADE2*, *ssa1*::*Kan*, *ssa2*::*HIS3*, *ssa3*::*TRP1*, *ssa4*:: *ura3-2f/pRS416P*_*GPD*_*-His*_*6*_*-SSA4*	This study
ASc822	*MATa*, *P*_*DAL5*_::*ADE2*, *ssa1*::*Kan*, *ssa2*::*HIS3*, *ssa3*::*TRP1*, *ssa4*:: *ura3-2f/pRS412P*_*P82*_*- SSA2*	This study
ASc823	*MATa*, *P*_*DAL5*_::*ADE2*, *ssa1*::*Kan*, *ssa2*::*HIS3*, *ssa3*::*TRP1*, *ssa4*:: *ura3-2f/pRS412P*_*P82*_*-SSA3*	This study

### Plasmids

The plasmids used in this study are listed in Table 2 [65]. pRS315P_SSA2_-SSA1/SSA2/SSA3/SSA4 is CEN and *LEU2* based plasmid that encodes Ssa1/Ssa2/Ssa3/Ssa4 respectively under same constitutive Ssa2 promoter. The Ssa Hsp70 based hybrids were constructed using PCR-based standard recombinant-DNA technology. For constructing *SSA23*, the regions of the gene encoding the NBD (amino acids 1–383) and the SBD (amino acids 385–649) were amplified using SSA2 or SSA3, respectively, as parental DNA templates. The PCR-amplified DNA encoding NBD and SBD contained 40-bp overlapping regions at 3´ and 5´ ends, respectively. The amplified PCR products were gel purified, combined together, and used as a template for overlap PCR with forward and reverse primers encoding NdeI and SphI restriction sites, respectively. The amplified overlap product was digested with NdeI and SphI, and ligated into pRS315-Ssa2, digested with the same restriction enzymes. The ligated plasmid (pRS315P_SSA2_-Ssa23) was confirmed using restriction digestion and sequencing of the gene encoding Ssa23. A plasmid encoding Ssa32 (pRS315P_SSA2_-Ssa32) was similarly constructed.

**Table 2:** List of plasmids.

Plasmid	Marker	Reference
pRS416P_GPD_-SYN	URA3	This study
pRS426P_GPD_-SYN	URA3	This study
pRS416P_GPD_-SYN(A30P)	URA3	This study
pRS416P_GPD_-SYN(E46K)	URA3	This study
pRS416P_GPD_-SYN(A53T)	URA3	This study
pRS426P_GPD_-SYN(A30P)	URA3	This study
pRS426P_GPD_-SYN(E46K)	URA3	This study
pRS426P_GPD_-SYN(A53T)	URA3	This study
pRS416P_GAL_-SYN	URA3	This study
pRS315P_SSA2_-SSA1	LEU2	Sharma & Masison, 2008
pRS315P_SSA2_-SSA2	LEU2	Sharma & Masison, 2008
pRS315P_SSA2_-SSA3	LEU2	Sharma & Masison, 2008
pRS315P_SSA2_-SSA4	LEU2	Sharma & Masison, 2008
pRS315P_SSA2_-SSA32	LEU2	This study
pRS315P_SSA2_-SSA23	LEU2	This study
pRS416P_GPD_-HTV-SSA1	URA3	This study
pRS416P_GPD_-HTV-SSA2	URA3	This study
pRS416P_GPD_-HTV-SSA3	URA3	This study
pRS416P_GPD_-HTV-SSA4	URA3	This study
pRS316GFP-ATG8	URA3	Gift by Dr. Ravi Manjithaya
pRS316mCherry-ATG8	URA3	Gift by Dr. Ravi Manjithaya
pRS412P_GAL_-SYN-GFP	ADE2	This study
pRS422P_GAL_-SYN-GFP	ADE2	This study
pRS426P_MET25_-FLAG-72Q-CFP	URA3	Gift by Dr. Kausik Chakraborty
pRS426P_MET25_-FLAG-46Q-CFP	URA3	Gift by Dr. Kausik Chakraborty
pRS426P_GPD_-GFP	URA3	This study
pRS412P_P82_-SSA2	ADE2	This study
pRS412P_P82_-SSA3	ADE2	This study

The yeast plasmid encoding wt α-syn was constructed using standard DNA recombinant technology by PCR amplification of the gene encoding α-syn, followed by restriction digestion with BamHI and XhoI restriction endonucleases, and sub-cloned into desired plasmids digested with the same enzymes. The point mutations in the α-syn encoding gene were introduced using site-directed mutagenesis. For protein purification, yeast plasmids were constructed that encoded Ssa1, Ssa2, Ssa3, or Ssa4 with a 5´ cleavable His_6_-tag, followed by the TEV protease recognition motif.

### Media and growth conditions

YPAD media consisted of 1% Yeast Extract (BD, USA-212750), 2% Peptone (BD, USA-244620), 2% Dextrose (Fisher, USA-50-99-7), and 35 mg/L Adenine (Sigma, USA-A9126). Strains transformed with plasmid were grown in selective SD media containing 0.67% Yeast nitrogen base (BD, USA-233520), 2% Dextrose as a carbon source with required amino acids, 100 mg/L Adenine. For the galactose inducible system, SGal media containing 2% Galactose (Sigma, USA-G0625) and 2% Raffinose (Sigma, USA-R0250) as carbon sources was used. For induction, the cells were grown in SD media until log phase, washed three times with sterile distilled water, and sub-cultured in SGal media. For induction of autophagy, transformants were grown in selective SD media until mid-log phase, washed 3–4 times with sterile distilled water, and transferred into media lacking ammonium sulfate (Sigma, USA-A4915).

For the yeast transformation experiments, all required strains were grown in liquid media until mid-log phase (~0.8 O.D._600nm_). The cell density was normalized using O.D._600nm_, and approximately-equal number of cells were used for transformation. Approximately 500 ng of plasmid was transformed using the PEG/LiAc method. Similar conditions were maintained for all strains during transformation. The images of transformants shown are representative of the entire plate.

### Protein purification

For Ssa2 purification, strain Asc200 was grown in YPAD liquid media for 24 h. Cells were collected by centrifugation, and re-suspended into Buffer A (20 mM HEPES, 150 mM NaCl, 25 mM KCl, and 25 mM MgCl_2_, pH 7.4). Cells were lysed using glass beads followed by sonication. His_6_-Ssa2 was purified from the supernatant using cobalt based Talon metal affinity resin. The eluted fractions were incubated with ATP-Agarose resin (Sigma, USA-A2767) for 4–5 h. The bound protein was eluted with buffer A, containing 7 mM ATP and 1 mM DTT. The eluted Ssa2 was dialyzed against buffer A and stored at -80°C until use. The purity was confirmed on 10% SDS-PAGE. Ssa1, Ssa3 and Ssa4 were similarly purified as described for Ssa2.

### Immunoblot analysis

Cell lysis was carried out with glass beads, and the lysate fractionated into supernatant and pellet. The proteins were separated by SDS-PAGE and transferred onto polyvinylidene fluoride (PVDF) membranes. For detection of α-syn, anti-α-syn antibody (CST, USA-2642) was incubated (1:2000) at 4°C overnight. The primary antibodies anti-GFP (Thermo Scientific, USA-MA5-15256), anti-Pgk1 (Life Technologies, USA-459250), anti-FLAG (Sigma, USA-F3165), and anti-mCherry (BioVision, USA-5993-100) were diluted 1:5000 and incubated at 25°C for 2 h.

The expression levels of different Ssa isoforms in A1-A4 strains were examined using comparative analysis of immunoblots obtained from A1-A4 compared to purified Ssa Hsp70s. The A1-A4 cells were lysed and the Ssa Hsp70 levels probed using antibodies against Hsp70s (Enzo Life Sciences, USA-SPA-822F), as described above. As seen in the S1A Fig, bands of differing intensities were observed. To examine whether the difference was because of differential affinity of the anti-Hsp70 antibody, or expression level differences of the chaperones, the His_6_-tagged Ssa Hsp70 isoforms were purified and equal amounts probed with the anti-Hsp70 antibodies, or anti-His_6_ antibodies (Pierce, USA-MA1-21315) as loading control. Similar differences in band intensity, as seen above with A1-A4 lysates, suggests that Hsp70 isoforms were expressed at similar levels in these strains.

### Dot blot analysis

Strains A1-A4 expressing Flag-Htt-72Q-CFP through a methionine repressible promoter were grown to mid-log phase in the presence of 1 mM methionine. Cells were washed and grown in media lacking methionine for 12 h. Cells were pelleted, lysed using glass beads, and equal amounts of protein (15 μg) were spotted onto activated PVDF membrane. The membrane was probed with anti-FLAG antibody, using procedures described above for the immunoblotting analysis.

### Confocal microscopy

For monitoring the co-localization of α-syn and autophagosomes, GFP-tagged α-syn and mCherry-tagged Atg8 were used. Cells co-transformed with both plasmids were grown in selective SD media until log phase, induced in SGal media, and mounted onto 1% Agarose blocks. Imaging was carried out at 100X/oil objective, with a numerical aperture of 1.45. The GFP and mCherry were excited with an argon laser and a helium-neon laser (GFP: 488 nm excitation; DsRed: 559 nm excitation) using a Nikon A1 plus Ti confocal microscope with a Nikon A1R scan head. Images were captured using NIS elements software. At least 200 cells expressing α-syn-GFP were analyzed.

### Intracellular glutathione assay

Glutathione levels (total GSH and its oxidized form GSSG) were analyzed as previously described [66]. Briefly, cells were grown in YPAD liquid media until mid-log phase and lysed in the presence of 0.1% TritonX-100 and 0.6% sulfosalicyclic acid. The debris was removed and 20 μl of the lysate was added to 700 μl of buffer (1X PBS + 5 mM EDTA, pH 7.4) containing 50 μl of 5,5´-dithio-bis(2-nitrobenzoic acid) (DTNB; 0.6 mg/ml) and 50 μl of glutathione reductase (GR; 14 μl/ml stock prepared from 250 units/ml). The reaction mixture was incubated for 30 seconds followed by the addition of β-NADPH (0.6 mg/ml). The GSH concentrations were estimated by measuring TNB (5´-thio-2-nitrobenzoic acid) absorbance at 412 nm. To measure GSSG, free GSH present in the lysate was covalently bound to 2-vinyl pyridine with the excess 2-vinyl pyridine being neutralized using triethanolamine. GSSG was measured using methods similar to those described above for GSH. The GSH and GSSG concentrations were measured using standard curves produced from known concentrations of commercially obtained GSH and GSSG.

### Quantitative real-time PCR

Quantitative real-time PCR (qRT-PCR) was used to measure the mRNA transcripts of *ATG8* and *α-SYN*. Phoshphoglycerol kinase (*PGK1*) was selected as a reference gene. Total RNA was isolated from cell lysates using a HiPurA Yeast RNA Purification Kit (HiMedia, India-MB611) following the manufacturer’s protocol. The cDNA was prepared from the isolated RNA using a cDNA synthesis kit (Verso, Thermo Scientific, USA-AB1453B), and used as a template for qRT-PCR. The cDNA samples were amplified with a DyNAmoColorFlash SYBR green qPCR kit (Thermo scientific, USA-FNZ416L) using a Steponeplus Real time PCR system (Applied Biosystems).

### Significance test

The experiments were performed in triplicate at a minimum. Student’s paired *t*-test was used to compare the groups for significance. The p values are shown as follows: * p < 0.05, ** p < 0.01, *** p < 0.001, **** p < 0.0001, with a p < 0.05 considered statistically significant.

## Supporting information

S1 Fig Ssa1-Ssa4 were expressed at similar levels in strains A1-A4, respectively. **(A)** The indicated strains were grown until mid-log phase. Cells were lysed, and equal amounts of cellular lysate were loaded onto SDS-PAGE, and probed with anti-Hsp70 antibody. The lower panel (Loading control) shows Amido Black staining of blot membranes, which served as loading and protein transfer controls. Also, shown are the percentage Hsp70 signal intensity relative to Pgk1. **(B)** Equal amounts of the indicated purified His_6_-tagged Ssa Hsp70 was loaded and probed with anti-Hsp70 antibody (upper panel), or anti-His_6_ tag antibody (lower panel). Shown are the percentage Hsp70 signal intensity relative to that obtained from anti-His_6_ antibody. **(C)** Indicated amount of total protein from cellular lysates were loaded to examine the sensitivity of detection by α-Hsp70 antibody. Also, shown are the percentage Hsp70 signal intensity relative to Pgk1. **(D)** The fraction Hsp70 signal intensity obtained in Panel S1A relative to that obtained using purified respective Hsp70 isoforms in Panel S1B. **(E)** The mRNA was isolated from strains A1-A4, and converted to cDNA as mentioned before. Quantitation was performed by qRT-PCR using primers specific for Hsp70. Error bars represent standard error of replicates performed 3 times.
(TIF)

S2 Fig The modulation of the Hsp70 isoforms expression does not alter α-syn associated toxicity. A2 and A3 strains (expressing Ssa Hsp70 isoforms from Ssa2 promoter (P_A2_)) were transformed with plasmids expressing Ssa2 and Ssa3 under Hsp82 promoter (P_82_) respectively. The resulting strains thus obtained were further transformed with CEN and 2μ plasmids expressing α-syn, and colony growth was monitored. **(A)** Growth phenotype of different strains onto solid media after incubation at 30°C for 5 days. As seen, only cells expressing Ssa3 from P_A2_ or P_82_ or both promoter show reduced α-syn toxicity. **(B)** The indicated strains were grown in liquid selective growth media until mid-log phase. The cells were lysed and the lysate was examined on immunoblot with anti-Hsp70 antibody.
(TIF)

S3 Fig α-syn associated toxicity was lower in strain A3 than in strain A2. **(A)** A2 and A3 strains were transformed with either empty plasmid (EV), or galactose regulatable α-syn expression CEN-based plasmid. Cells were grown in liquid selective SD media overnight, washed with sterile H_2_O, serially diluted, and cultured onto solid SD, or SGal media. Shown is growth after incubation at 30°C for 5 days. **(B)** Strains A2 and A3 were transformed with either empty plasmid (EV) or galactose regulatable α-syn-GFP expression plasmid. Cells were grown in liquid selective SD media overnight, washed with sterile H_2_O, and induced for 24 h in SGal media before being serially diluted and plated onto solid SD or SGal media. Shown is the growth after 3 or 4 days of incubation at 30°C.
(TIF)

S4 Fig α-syn was expressed at similar levels in WT, A2 and A3. **(A)** wt cells harboring EV or p426-P_GPD_-α-syn were serially diluted and cultured on solid SD media lacking uracil. **(B)** wt cells transformed with 2μ plasmid encoding α-syn under GPD promoter were processed for immunoblotting with anti α-syn antibody. **(C)** wt, A2, and A3 cells transformed with a CEN-based plasmid encoding α-syn under a GPD promoter, were processed for immunoblotting with an anti α-syn antibody. Immunostaining with an anti-Pgk1 antibody, and Amido Black staining were used as loading controls.
(TIF)

S5 Fig GPD promoter-driven GFP was expressed at similar levels in strain A2 and strain A3. The strains were transformed with 2μ plasmid encoding GFP under a GPD promoter. The pool of 5–6 transformants was grown in liquid SD media lacking uracil. Cells were lysed, and the cell lysates probed with antibody against GFP, or Pgk1 (internal control). The lower panel shows the same blot, stained with Amido Black. As seen, GFP was found to be at similar levels in both strain A2 and strain A3.
(TIF)

S6 Fig A3 and A4 reduced toxicity associated with the accumulation of 72Q. **(A)** WT cells harboring EV or p426P_MET25_-FLAG-htt-72Q-CFP were grown in presence of methionine upto mid-log phase, serially diluted and cultured on solid SD media lacking uracil. **(B)** Strains A1-A4 were transformed with p426P_MET25_-FLAG-htt-72Q-CFP, a plasmid encoding 72Q under a methionine responsive promoter (72Q), or p426 (EV). A total of 5–6 transformants were pooled, grown in liquid SD media, serially diluted and cultured on solid SD media lacking uracil. **(C)** Relative abundance of FLAG-htt-72Q-CFP in strains A1-A4, using dot-blot analysis. The assay was performed as described in Materials and Methods. Shown is the image acquired after 0.2 min (upper panel) and 1 min (lower panel). **(D)** Quantitation was performed by qRT-PCR using primers specific for CFP. Error bars represent the standard error of replicates performed 3 times.
(TIF)

S7 Fig A32 grew slower compared to other strains. Indicated strains were grown in liquid YPAD media and the growth was monitored as increased optical density (O.D._600nm_) over time. As shown, among the four strains examined, strain A32 grew slowest.
(TIF)

S8 Fig Autophagy inhibition enhanced growth defects by α-syn mutants in strain A3. The growth phenotype of *atg5Δ* cells expressing α-syn mutants was monitored as described for wt α-syn in Fig 5A. As shown, strain A3 defective for autophagy grew poorly upon expression of disease-associated α-syn mutants.
(TIF)

S9 Fig Autophagy induction under nitrogen starvation conditions. The wt cells transformed with a plasmid encoding GFP-Atg8, were grown until mid-log phase. Cells were subsequently sub-cultured in liquid SD media, with or without ammonium sulfate. Cells were collected at the indicated times, and equal amounts of cellular lysate were probed with anti-GFP antibody. Anti-Pgk1 antibody and Amido Black staining were used as a loading controls. The increased levels over time of free GFP under nitrogen starvation conditions suggests activation of autophagy.
(TIF)

S10 Fig ATG8 level increased upon α-syn induction in the wild type (wt) strain. The wt strain, transformed with empty plasmid (EV), or with a plasmid encoding galactose driven α-syn were grown in inducible media for 12 h. Quantitation by qRT-PCR was performed using primers specific for *ATG8* or *PGK1* (internal control). As seen, α-syn increased *ATG8* transcription by more than 3 fold as compared to empty vector (EV) alone. Error bars represent the standard error of replicates performed 3 times. P-value was calculated using paired *t*-test.
(TIF)

S11 Fig mCherry-Atg8 is not degraded in strains lacking Atg5. *atg5Δ*/A2 and *atg5Δ*/A3 strains were co-transformed with pRS316-mCherry-ATG8 and p412P_GAL_-SYN-GFP (CEN) or p422P_GAL_-SYN-GFP (2μ). Transformants were grown in inducing conditions for 12 hours before processing for western blotting against anti-mCherry antibody. α-Pgk1 was used as internal control.
(TIF)

S12 Fig Increased number of α-syn-GFP puncta were observed in *atg5Δ*/A3 cells compared to A3 cells. The cells were grown as described in Fig 7. **(A)** Confocal microscopy images of representative *atg5Δ*/A3 cells. **(B)** The frequency of cells showing α-syn-GFP puncta in *atg5Δ*/A3 and A3 cells. Data for A3 cells was used from Fig 7B.
(TIF)

S13 Fig Ssa3 strain showed higher intracellular GSH levels. Glutathione levels were measured using the DTNB method as described in the Materials and Methods. The indicated strains were harvested in mid-log phase, and lysed in the presence of sulfosalicylic acid. The total protein in cell lysate was measured, and an equal amount of protein was used for glutathione estimation. Shown are the total reduced levels (GSH), as well as the ratio of reduced GSH to oxidized glutathione levels (GSH/GSSG). P-values were calculated using paired *t*-test (p-value = 1.5*10^−4^)
(TIF)
