# Supplementary figures and images for: The yeast stress inducible Ssa Hsp70 reduces α-synuclein toxicity by promoting its degradation through autophagy
Source: PLoS Genet. 2018 Oct 30;14(10):e1007751. doi: 10.1371/journal.pgen.1007751 (PMC6226208; doi:10.1371/journal.pgen.1007751)

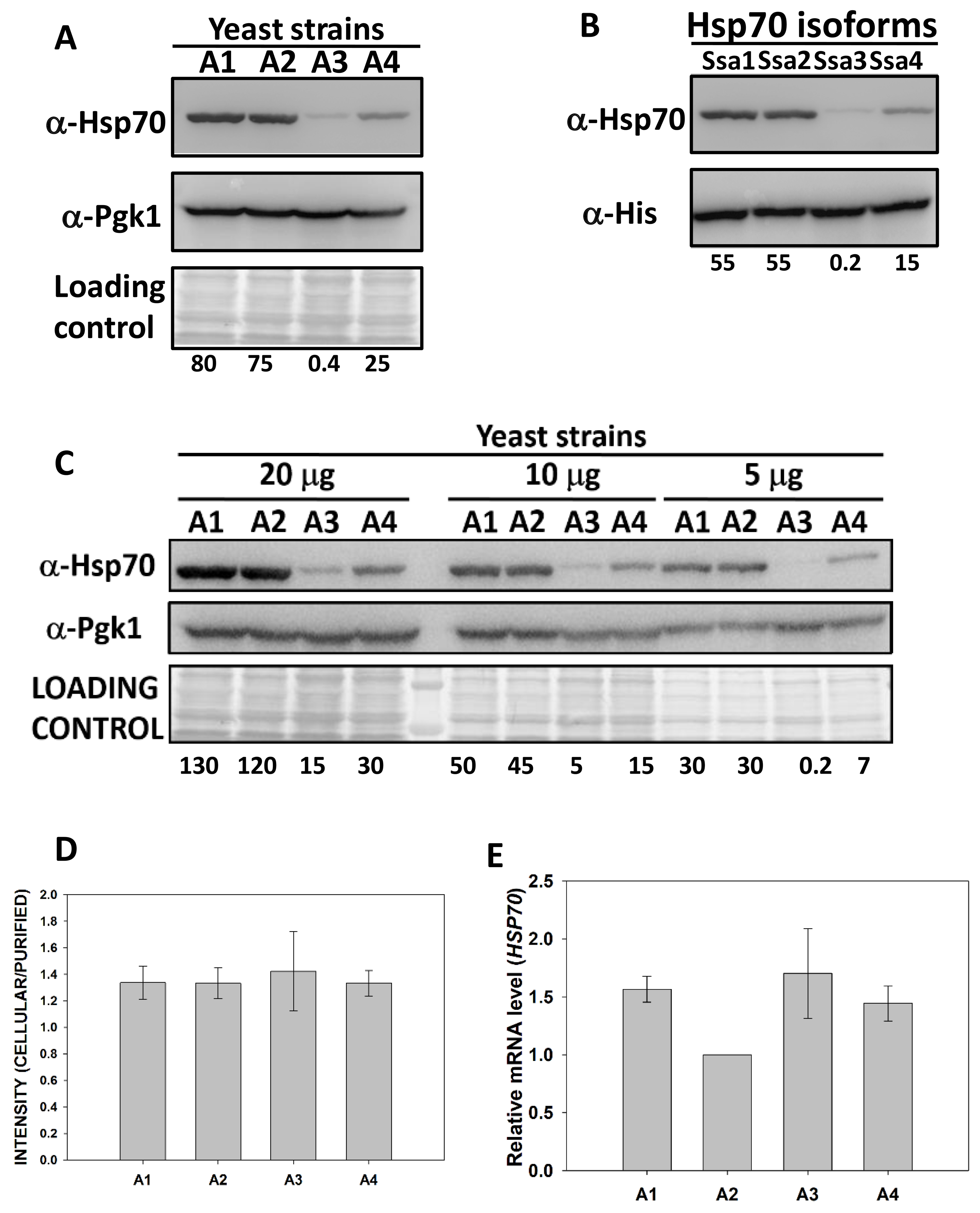

Supplement: S1 Fig — (A) The indicated strains were grown until mid-log phase. Cells were lysed, and equal amounts of cellular lysate were loaded onto SDS-PAGE, and probed with anti-Hsp70 antibody. The lower panel (Loading control) shows Amido Black staining of blot membranes, which served as loading and protein transfer controls. Also, shown are the percentage Hsp70 signal intensity relative to Pgk1. (B) Equal amounts of the indicated purified His6-tagged Ssa Hsp70 was loaded and probed with anti-Hsp70 antibody (upper panel), or anti-His6 tag antibody (lower panel). Shown are the percentage Hsp70 signal intensity relative to that obtained from anti-His6 antibody. (C) Indicated amount of total protein from cellular lysates were loaded to examine the sensitivity of detection by α-Hsp70 antibody. Also, shown are the percentage Hsp70 signal intensity relative to Pgk1. (D) The fraction Hsp70 signal intensity obtained in Panel S1A relative to that obtained using purified respective Hsp70 isoforms in Panel S1B. (E) The mRNA was isolated from strains A1-A4, and converted to cDNA as mentioned before. Quantitation was performed by qRT-PCR using primers specific for Hsp70. Error bars represent standard error of replicates performed 3 times. (TIF) [file pgen.1007751.s001.tif]

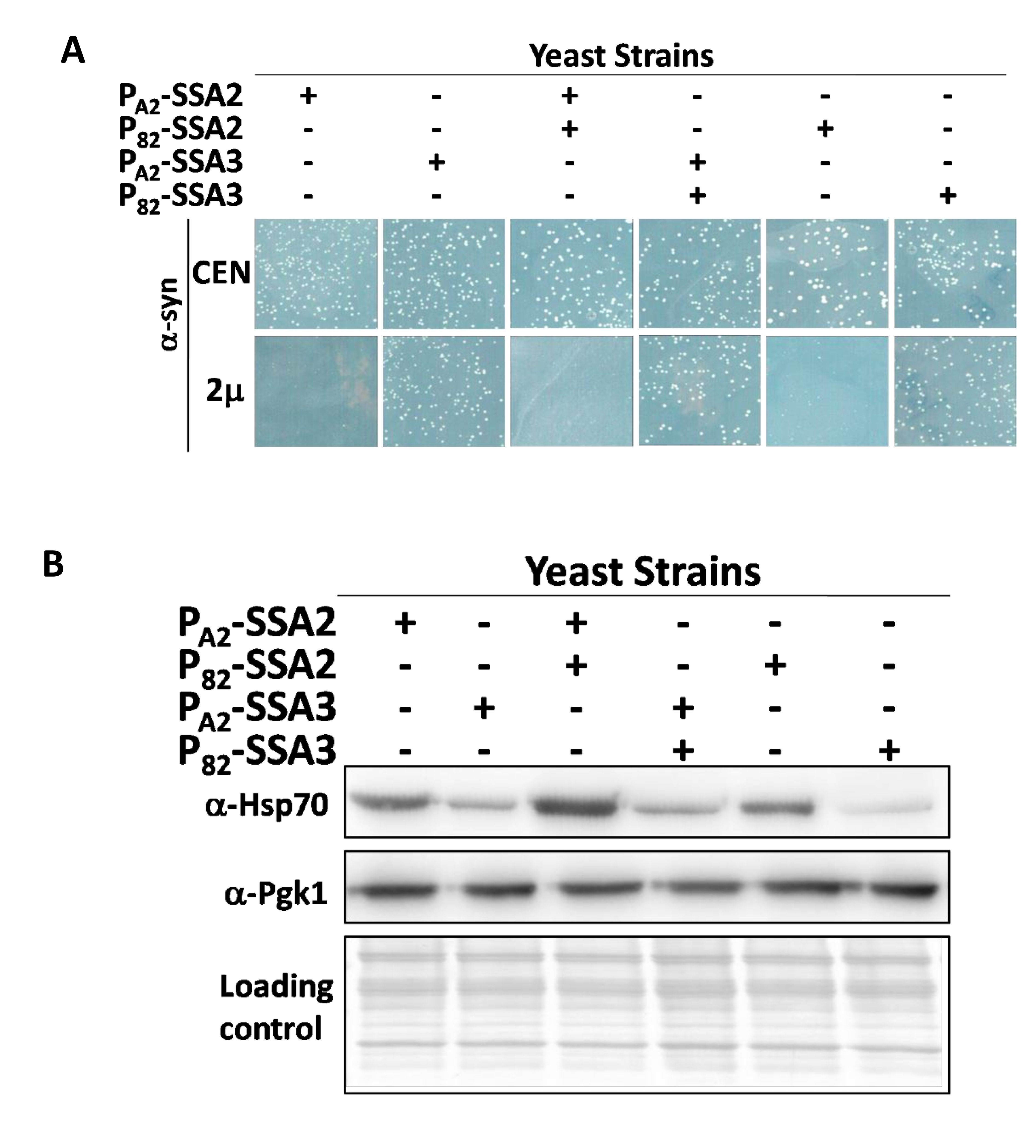

Supplement: S2 Fig — A2 and A3 strains (expressing Ssa Hsp70 isoforms from Ssa2 promoter (PA2)) were transformed with plasmids expressing Ssa2 and Ssa3 under Hsp82 promoter (P82) respectively. The resulting strains thus obtained were further transformed with CEN and 2μ plasmids expressing α-syn, and colony growth was monitored. (A) Growth phenotype of different strains onto solid media after incubation at 30°C for 5 days. As seen, only cells expressing Ssa3 from PA2 or P82 or both promoter show reduced α-syn toxicity. (B) The indicated strains were grown in liquid selective growth media until mid-log phase. The cells were lysed and the lysate was examined on immunoblot with anti-Hsp70 antibody. (TIF) [file pgen.1007751.s002.tif]

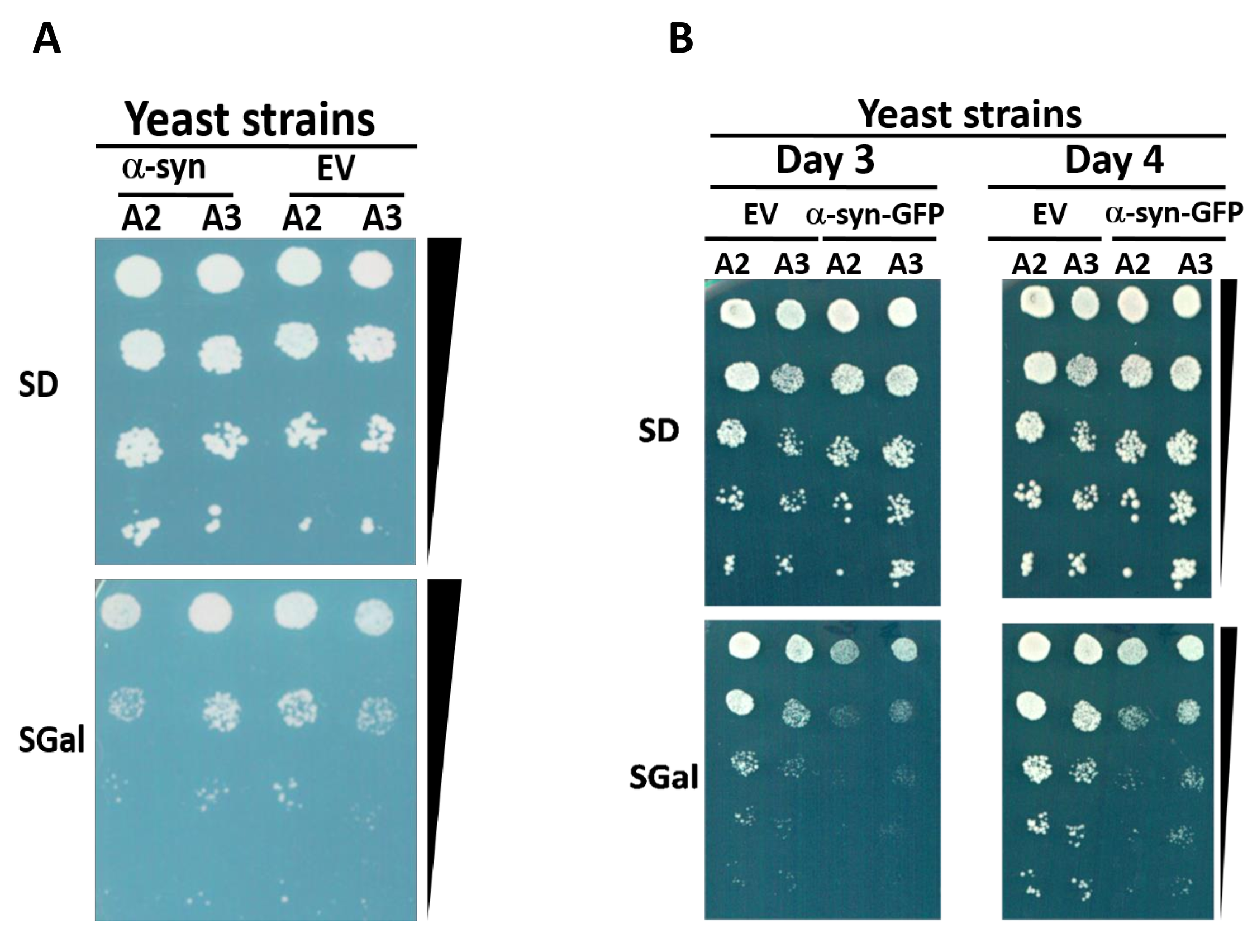

Supplement: S3 Fig — (A) A2 and A3 strains were transformed with either empty plasmid (EV), or galactose regulatable α-syn expression CEN-based plasmid. Cells were grown in liquid selective SD media overnight, washed with sterile H2O, serially diluted, and cultured onto solid SD, or SGal media. Shown is growth after incubation at 30°C for 5 days. (B) Strains A2 and A3 were transformed with either empty plasmid (EV) or galactose regulatable α-syn-GFP expression plasmid. Cells were grown in liquid selective SD media overnight, washed with sterile H2O, and induced for 24 h in SGal media before being serially diluted and plated onto solid SD or SGal media. Shown is the growth after 3 or 4 days of incubation at 30°C. (TIF) [file pgen.1007751.s003.tif]

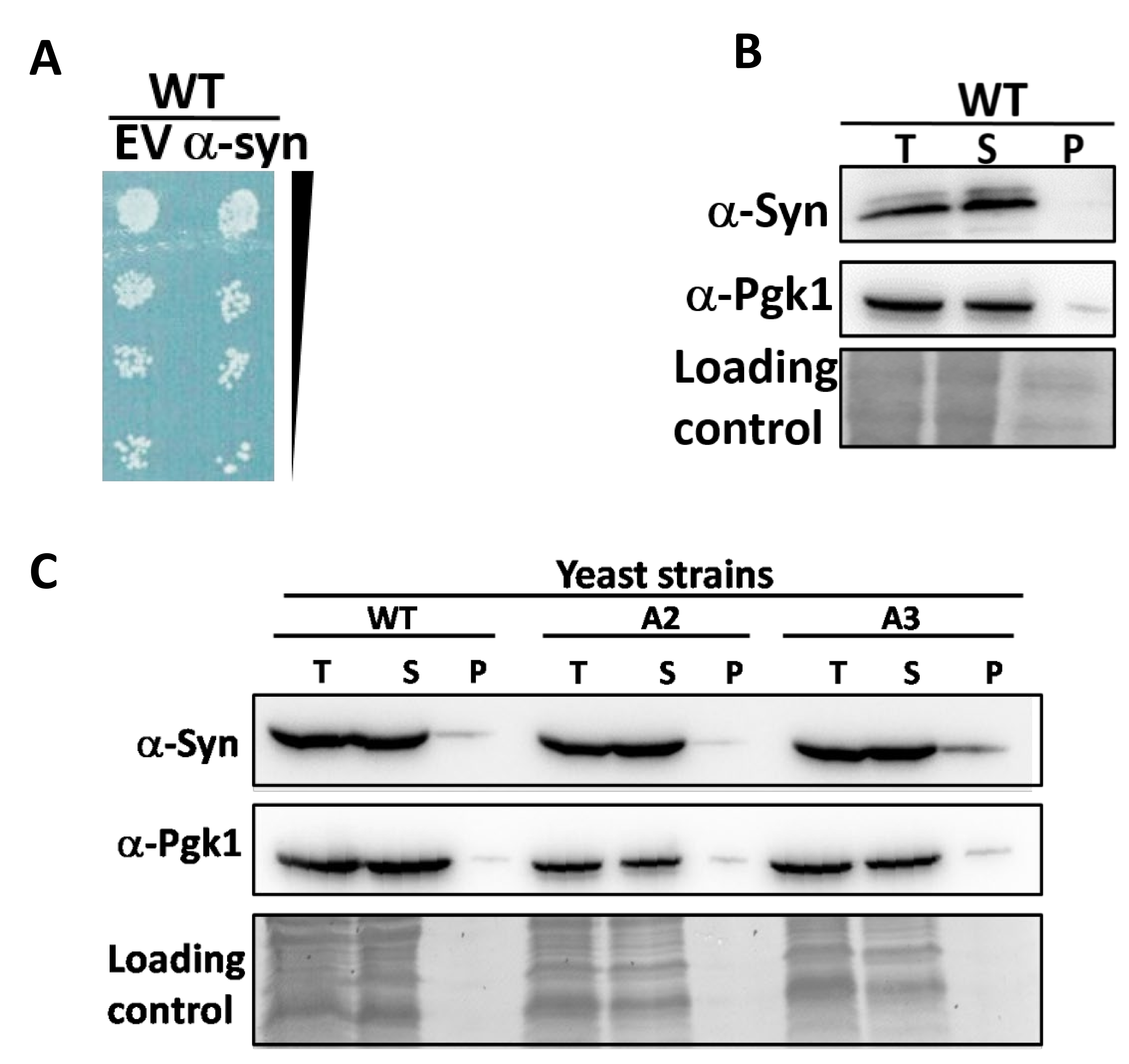

Supplement: S4 Fig — (A) wt cells harboring EV or p426-PGPD-α-syn were serially diluted and cultured on solid SD media lacking uracil. (B) wt cells transformed with 2μ plasmid encoding α-syn under GPD promoter were processed for immunoblotting with anti α-syn antibody. (C) wt, A2, and A3 cells transformed with a CEN-based plasmid encoding α-syn under a GPD promoter, were processed for immunoblotting with an anti α-syn antibody. Immunostaining with an anti-Pgk1 antibody, and Amido Black staining were used as loading controls. (TIF) [file pgen.1007751.s004.tif]

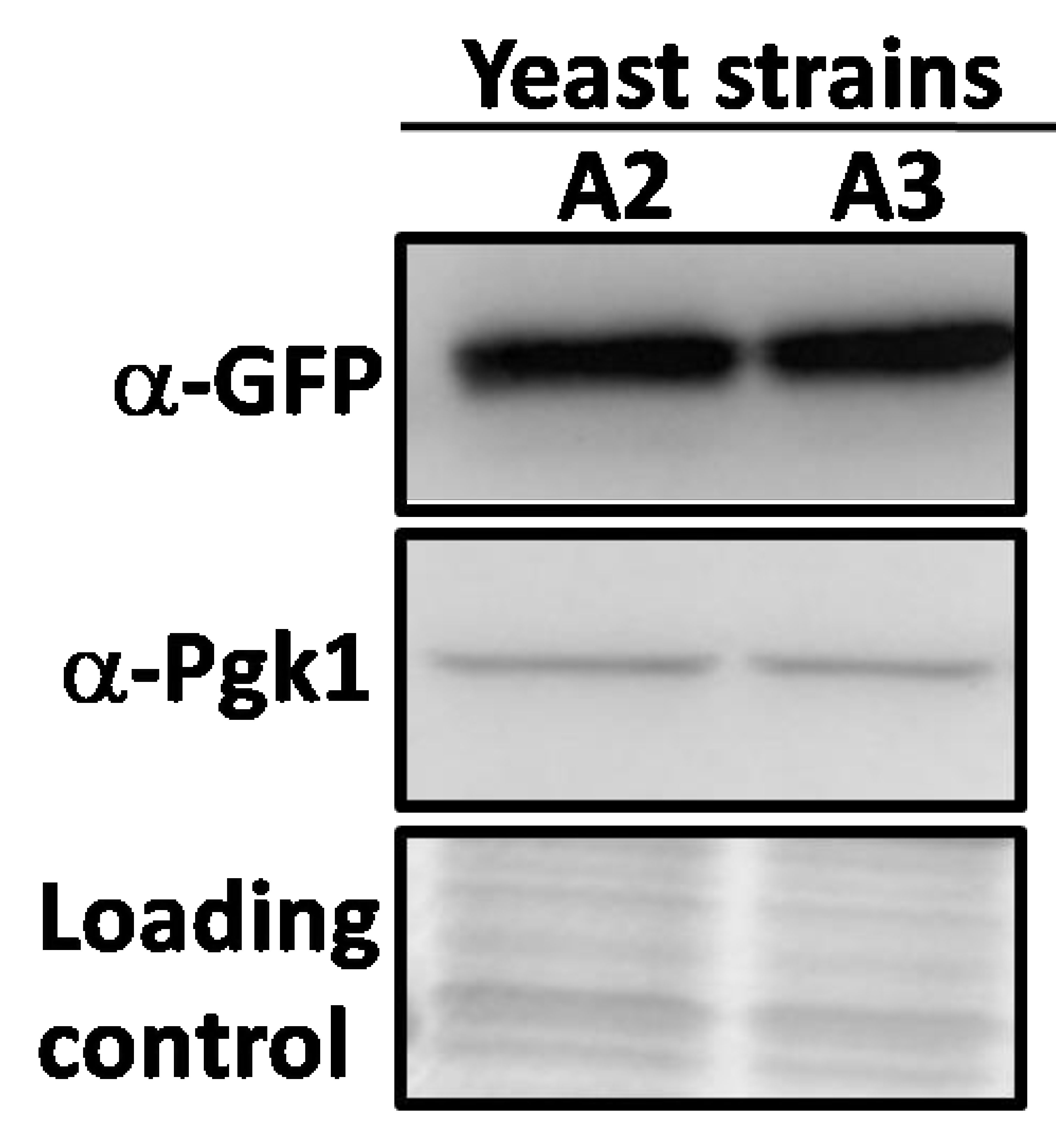

Supplement: S5 Fig — The strains were transformed with 2μ plasmid encoding GFP under a GPD promoter. The pool of 5–6 transformants was grown in liquid SD media lacking uracil. Cells were lysed, and the cell lysates probed with antibody against GFP, or Pgk1 (internal control). The lower panel shows the same blot, stained with Amido Black. As seen, GFP was found to be at similar levels in both strain A2 and strain A3. (TIF) [file pgen.1007751.s005.tif]

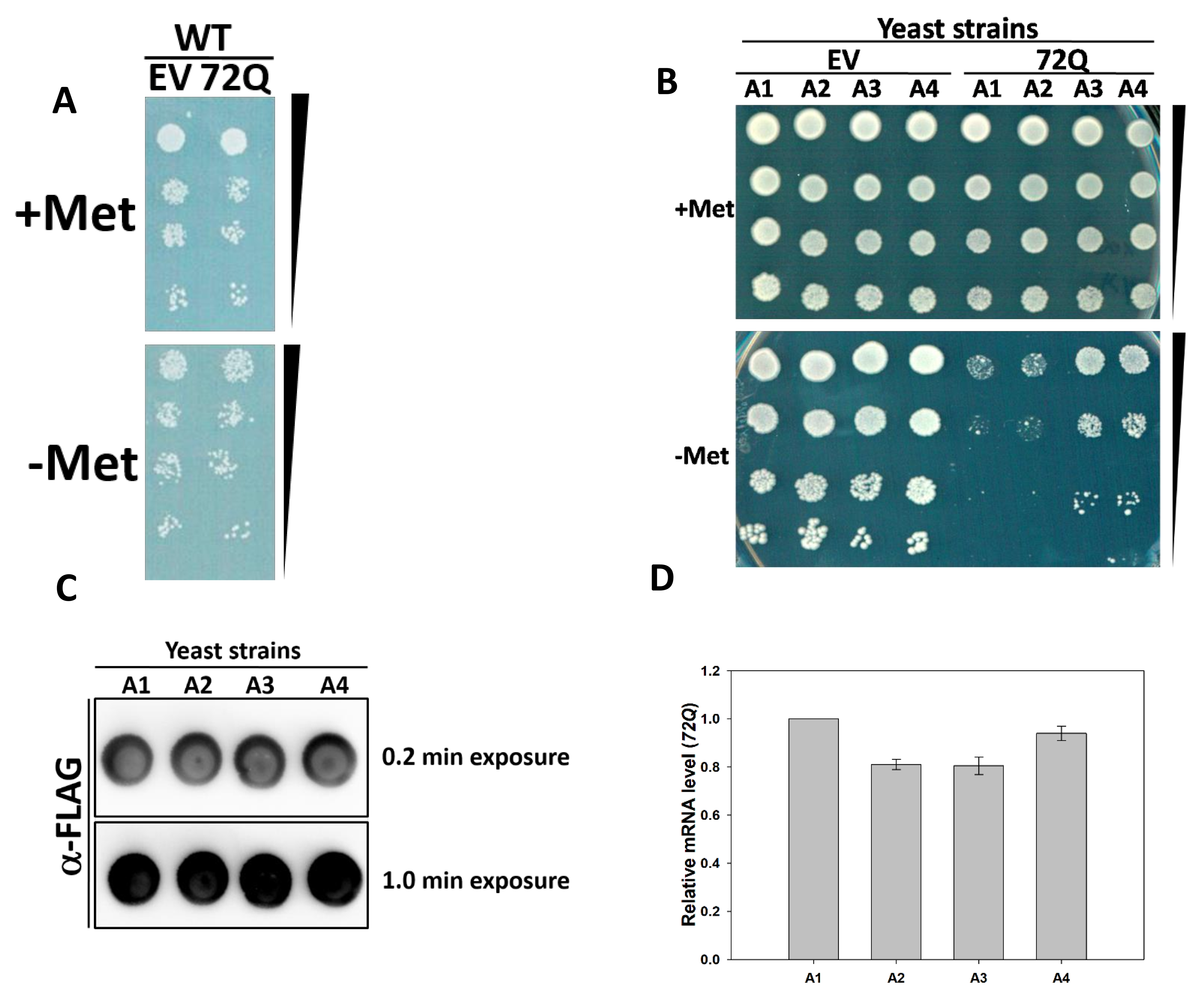

Supplement: S6 Fig — (A) WT cells harboring EV or p426PMET25-FLAG-htt-72Q-CFP were grown in presence of methionine upto mid-log phase, serially diluted and cultured on solid SD media lacking uracil. (B) Strains A1-A4 were transformed with p426PMET25-FLAG-htt-72Q-CFP, a plasmid encoding 72Q under a methionine responsive promoter (72Q), or p426 (EV). A total of 5–6 transformants were pooled, grown in liquid SD media, serially diluted and cultured on solid SD media lacking uracil. (C) Relative abundance of FLAG-htt-72Q-CFP in strains A1-A4, using dot-blot analysis. The assay was performed as described in Materials and Methods. Shown is the image acquired after 0.2 min (upper panel) and 1 min (lower panel). (D) Quantitation was performed by qRT-PCR using primers specific for CFP. Error bars represent the standard error of replicates performed 3 times. (TIF) [file pgen.1007751.s006.tif]

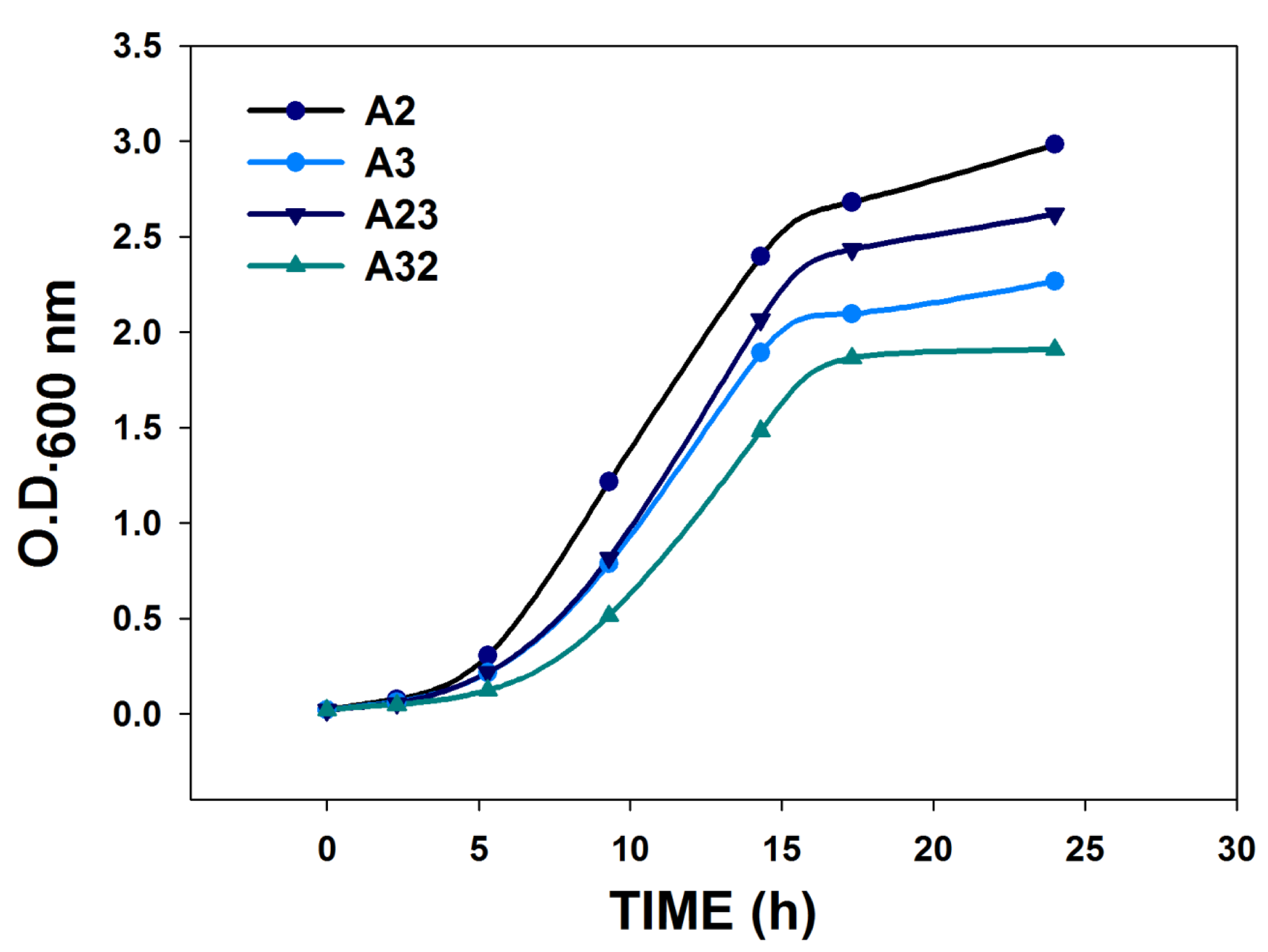

Supplement: S7 Fig — Indicated strains were grown in liquid YPAD media and the growth was monitored as increased optical density (O.D.600nm) over time. As shown, among the four strains examined, strain A32 grew slowest. (TIF) [file pgen.1007751.s007.tif]

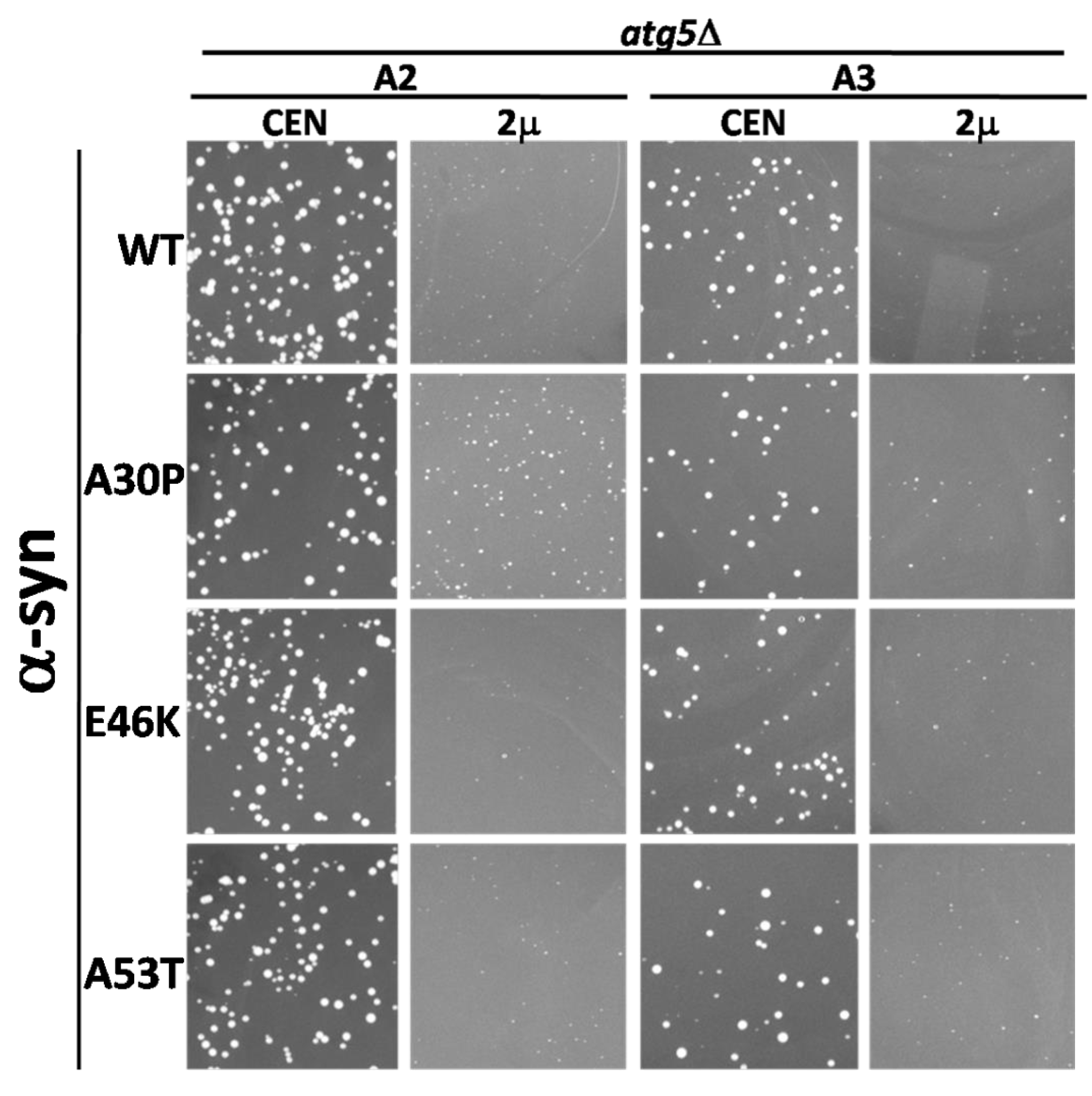

Supplement: S8 Fig — The growth phenotype of atg5Δ cells expressing α-syn mutants was monitored as described for wt α-syn in Fig 5A. As shown, strain A3 defective for autophagy grew poorly upon expression of disease-associated α-syn mutants. (TIF) [file pgen.1007751.s008.tif]

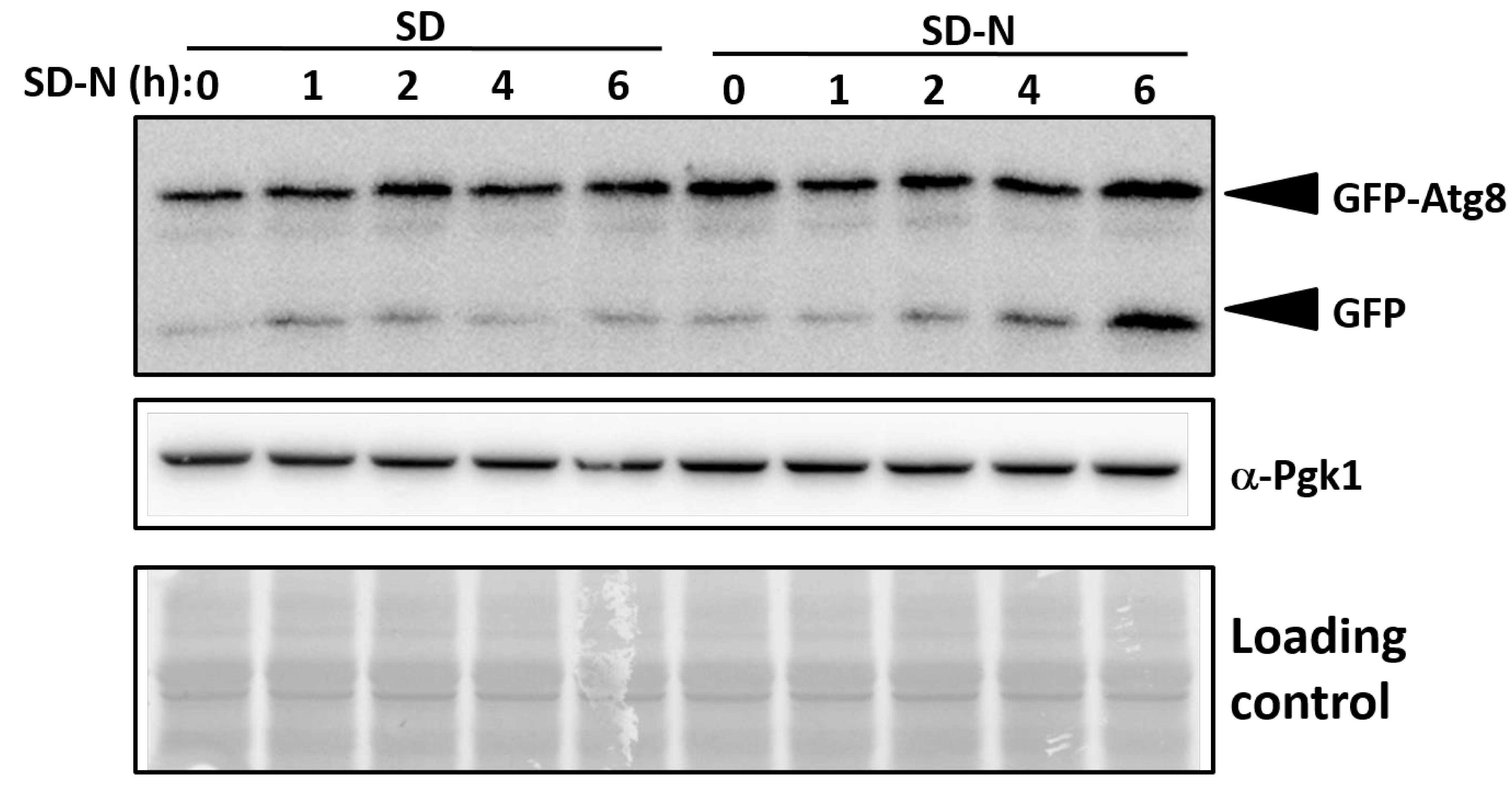

Supplement: S9 Fig — The wt cells transformed with a plasmid encoding GFP-Atg8, were grown until mid-log phase. Cells were subsequently sub-cultured in liquid SD media, with or without ammonium sulfate. Cells were collected at the indicated times, and equal amounts of cellular lysate were probed with anti-GFP antibody. Anti-Pgk1 antibody and Amido Black staining were used as a loading controls. The increased levels over time of free GFP under nitrogen starvation conditions suggests activation of autophagy. (TIF) [file pgen.1007751.s009.tif]

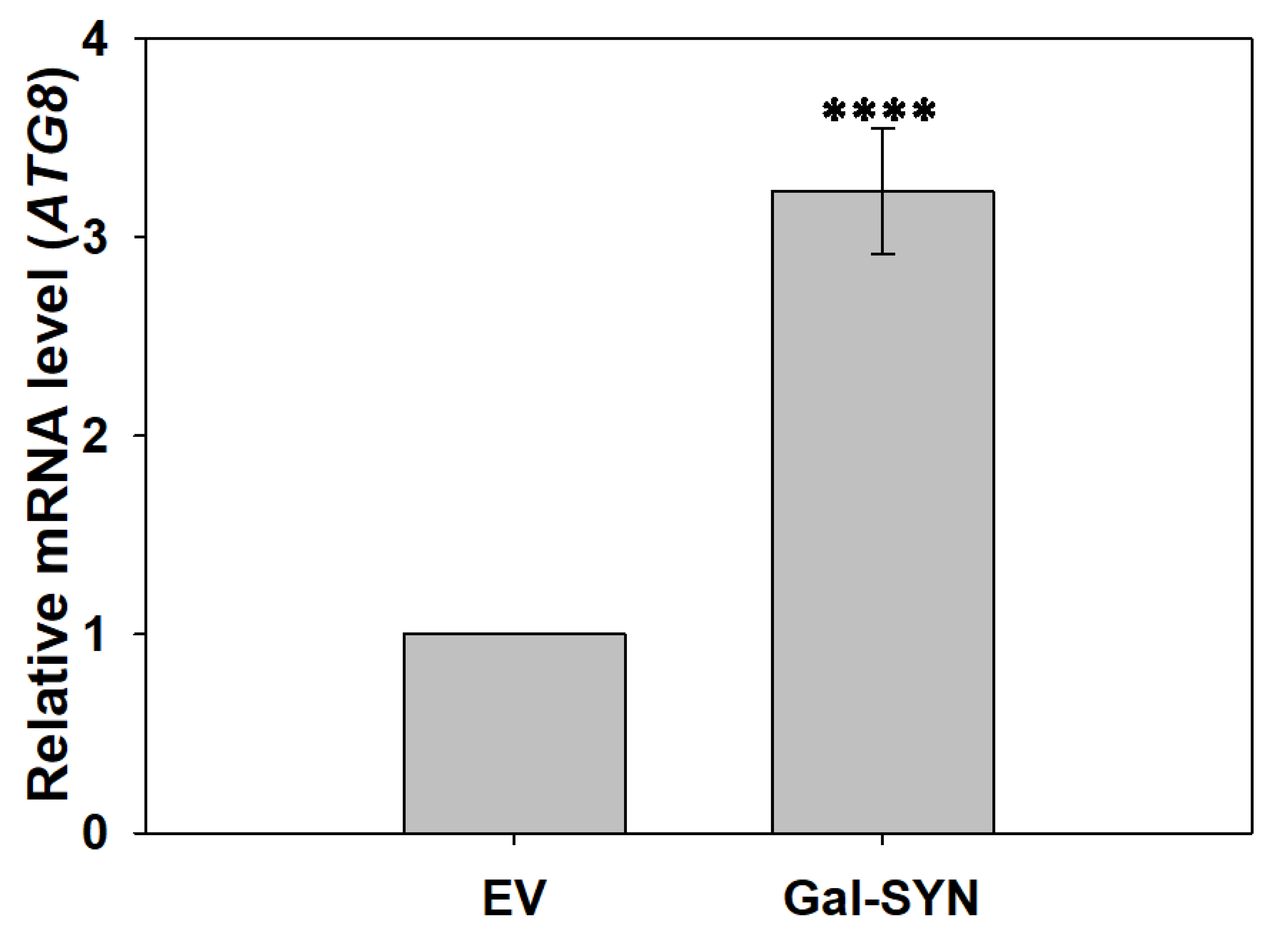

Supplement: S10 Fig — The wt strain, transformed with empty plasmid (EV), or with a plasmid encoding galactose driven α-syn were grown in inducible media for 12 h. Quantitation by qRT-PCR was performed using primers specific for ATG8 or PGK1 (internal control). As seen, α-syn increased ATG8 transcription by more than 3 fold as compared to empty vector (EV) alone. Error bars represent the standard error of replicates performed 3 times. P-value was calculated using paired t-test. (TIF) [file pgen.1007751.s010.tif]

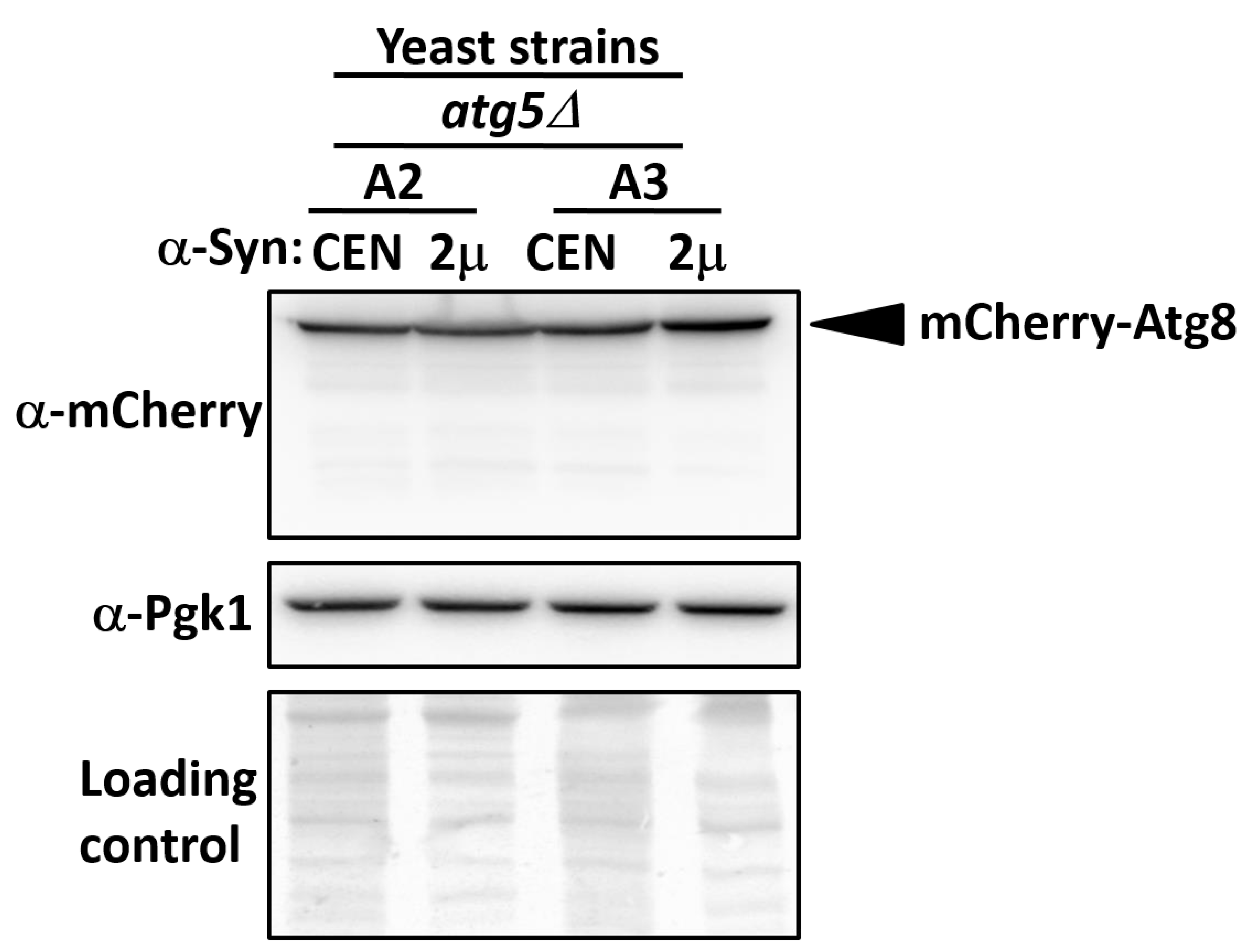

Supplement: S11 Fig — atg5Δ/A2 and atg5Δ/A3 strains were co-transformed with pRS316-mCherry-ATG8 and p412PGAL-SYN-GFP (CEN) or p422PGAL-SYN-GFP (2μ). Transformants were grown in inducing conditions for 12 hours before processing for western blotting against anti-mCherry antibody. α-Pgk1 was used as internal control. (TIF) [file pgen.1007751.s011.tif]

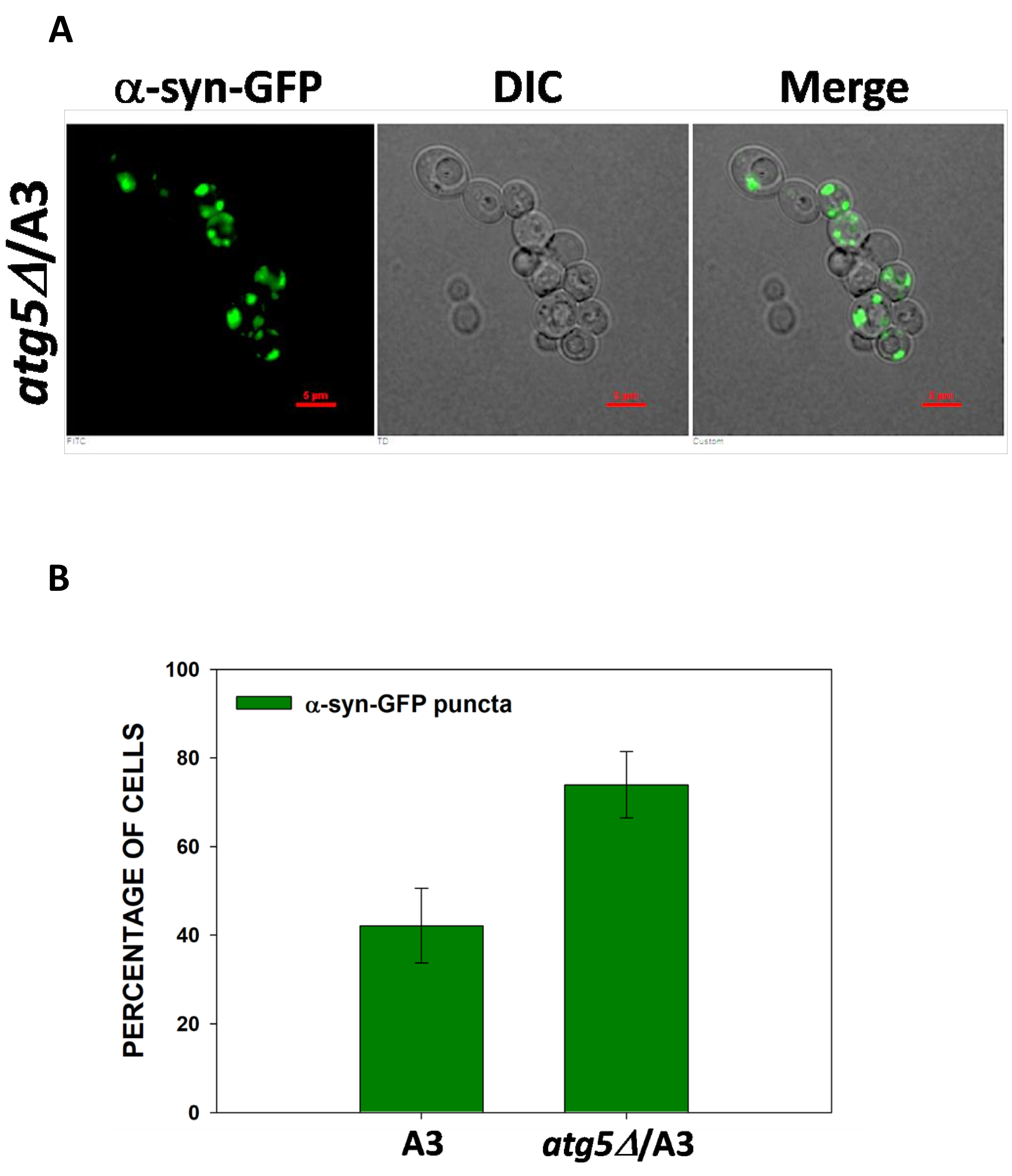

Supplement: S12 Fig — The cells were grown as described in Fig 7. (A) Confocal microscopy images of representative atg5Δ/A3 cells. (B) The frequency of cells showing α-syn-GFP puncta in atg5Δ/A3 and A3 cells. Data for A3 cells was used from Fig 7B. (TIF) [file pgen.1007751.s012.tif]

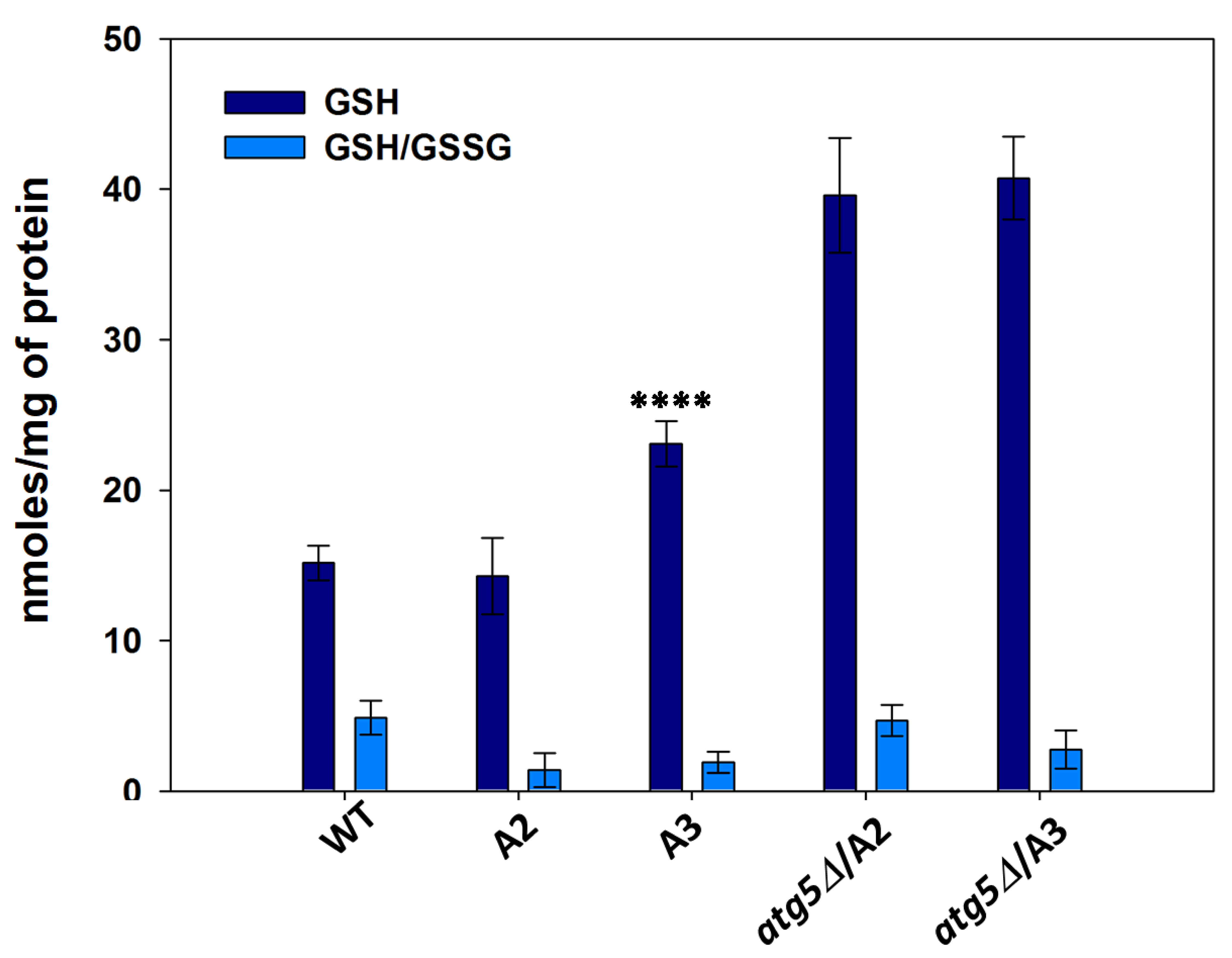

Supplement: S13 Fig — Glutathione levels were measured using the DTNB method as described in the Materials and Methods. The indicated strains were harvested in mid-log phase, and lysed in the presence of sulfosalicylic acid. The total protein in cell lysate was measured, and an equal amount of protein was used for glutathione estimation. Shown are the total reduced levels (GSH), as well as the ratio of reduced GSH to oxidized glutathione levels (GSH/GSSG). P-values were calculated using paired t-test (p-value = 1.5*10−4) (TIF) [file pgen.1007751.s013.tif]
